# Neuroanatomical Mapping of Gerbil Corticostriatal and Thalamostriatal Projections Reveals the Parafascicular Nucleus as a Relay for Vestibular Information to the Entire Striatum

**DOI:** 10.1523/ENEURO.0246-24.2025

**Published:** 2025-03-07

**Authors:** Jared B. Smith, Sean S. Hong, Damian J. Murphy, Evelynne Dangcil, Jacqueline Nacipucha, Aaron Tucker, Nicolas L. Carayannopoulos, Mina Beshy, Shrivaishnavi Chandrasekar, Eran Peci, Matthew Y. Kiel, P. Ashley Wackym, Justin D. Yao, Todd M. Mowery

**Affiliations:** ^1^Molecular Neurobiology Laboratory, Salk Institute for Biological Studies, La Jolla, California 92037; ^2^Department of Otolaryngology – Head and Neck Surgery, Rutgers Robert Wood Johnson Medical School, New Brunswick, New Jersey 08854; ^3^Rutgers Brain Health Institute, New Brunswick, New Jersey 08854

**Keywords:** corticostriatal, parafascicular nucleus, striatum, thalamostriatal, vestibular dysfunction, vestibular

## Abstract

The striatum is the primary input nucleus of the basal ganglia, integrating a dense plexus of inputs from the cerebral cortex and thalamus to regulate action selection and learning. Neuroanatomical mapping of the striatum and its subcompartments has been carried out extensively in rats and mice, nonhuman primates, and cats allowing comparative neuroanatomy studies to derive heuristics about striatal composition and function. Here, we systematically map corticostriatal topography from motor, somatosensory, auditory, and visual cortices as well as thalamostriatal parafascicular (PfN) inputs in the Mongolian gerbil. We also map a pathway reported in mice from medial vestibular nucleus to the PfN that could convey vestibular information to the striatum. Our findings align with those of similar studies in other rodents, indicating homologous neuroanatomical connectivity patterns within the corticostriatal projectome across Rodentia. We observed corticostriatal peaks of dense labeling for each input with a diffuse projection throughout striatal subregions from each cortical region, suggesting a global integration of all cortical information by the striatum. Thalamostriatal projections from PfN covered most of the striatum with a peak of PfN-specific compartmentalized labeling similar to other sensory and motor systems. We also confirm the connection from the medial vestibular nucleus to PfN thalamus, indicating that vestibular information may be widely integrated throughout the striatum. The findings build upon our body of knowledge on striatal connectivity across mammalian species and provide a foundation for striatal research focusing on vestibulothalamostriatal circuits in Rodentia.

## Graphical abstract

**Figure:**
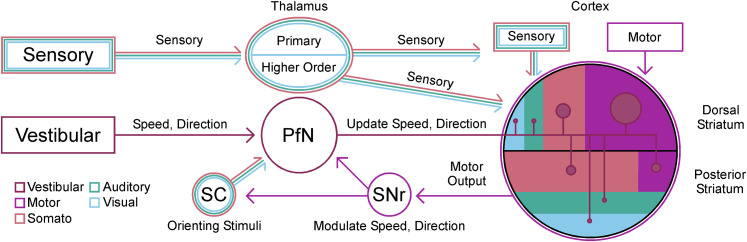
Visual Abstract

## Significance Statement

In this study, systematic mapping of the projections to striatum from motor, somatosensory, auditory, and visual cortices in Mongolian gerbil reveal commonalities with rodents. Principally, while some areas receive compartmentalized innervation from specific modalities, there also exists a global interspersed plexus of integrating inputs from each cortical area to each striatal subregion. We also demonstrate thalamostriatal innervation from parafascicular thalamus (PfN) that is homologous to other rodents and primates. Finally, we confirm a pathway from the medial vestibular nucleus to PfN thalamus that could broadly convey vestibular information across the striatum. Our results reveal common principles in neuroanatomical connectivity across another mammalian species and provide an anatomical map to guide future vestibular striatal studies in gerbils and other animal models.

## Introduction

The striatum is an important subcortical hub for sensory and motor integration (Alloway et al., 2006; Reig and Silberberg, 2014; Smith et al., 2022). It receives extensive glutamatergic inputs from cortical and thalamic projection neurons and is thought to integrate these diverse modalities to support its role in controlling action selection and learning (Doig et al., 2010; Huerta-Ocampo et al., 2014; Arber and Costa, 2022). Cortical projections to the striatum arise from pyramidal tract (PT) type neurons as well as ipsilateral and intratelencephalic tract (IT) neurons (Wilson, 1987; Reiner et al., 2010; Shepherd, 2013). These excitatory inputs drive striatal microcircuits composed of D1 and D2 medium spiny GABAergic projection neurons (MSNs), parvalbumin fast-spiking interneurons (PV), and cholinergic interneurons (ChAT; Klug et al., 2018; Gómez-Ocádiz and Silberberg, 2023), which in turn regulate downstream structures in the direct and indirect pathways (DeLong et al., 1984). Additional excitatory drive of striatal microcircuits arrives from a host of thalamic nuclei (Sadikot et al., 1992a,b; Harting et al., 2001; Smith et al., 2012a,b, 2014; Ellender et al., 2013; Wall et al., 2013; Alloway et al., 2014; Smith and Wichmann, 2015; Chen et al., 2019; Ponvert and Jaramillo, 2019). Of these, the parafascicular nucleus of the thalamus (PfN) sends the strongest projections to striatum, forming synapses on striatal neurons to modulate striatal output and facilitate sensory-response learning (Alloway et al., 2017).

Over the past few decades, numerous neuroanatomical tracing studies have been conducted to map the corticostriatal topography in a variety of species, typically using monosynaptic anterograde and retrograde tracing techniques to reveal the patterns of cortical input to the striatum (Hoffer and Alloway, 2001; Wright et al., 2001; Hoffer et al., 2005; Alloway et al., 2006, 2009; Pan et al., 2008; Mowery et al., 2011, 2017; Paraouty and Mowery, 2021; Pidoux et al., 2011; Shepherd, 2013; Reig and Silberberg, 2014, 2016; Zingg et al., 2014; Ponvert and Jaramillo, 2019; Foster et al., 2021; Smith et al., 2022). This body of work has shown common principles of striatal organization among rodents, nonhuman primates, and cats (Graybiel, 1984; Selemon and Goldman-Rakic, 1985; Donoghue and Herkenham, 1986; Ragsdale and Graybiel, 1988; Sadikot et al., 1992a,b; Kincaid and Wilson, 1996; Haber et al., 2000; McFarland and Haber, 2000; Voorn et al., 2004; Hintiryan et al., 2016; Hooks et al., 2018; Aoki et al., 2019; Borra et al., 2021; Smith et al., 2022). Using these approaches, researchers have divided the striatum into apparent functional subdivisions, which include the ventral striatum (e.g., nucleus accumbens core and shell), the dorsomedial striatum (DMS), dorsolateral striatum (DLS), and the posterior tail of the striatum (Voorn et al., 2004; Hintiryan et al., 2016; Hooks et al., 2018; Aoki et al., 2019). These subdivisions are functional nodes defined by the constellation of cortical regions that intersect in any specific three-dimensional region of the striatum (Hintiryan et al., 2016; Hunnicutt et al., 2016). This connectivity paradigm supports cross-modal integration of numerous types of relevant information (e.g., somatosensory and motor in DLS) to support cohesive integration, which is the basis for current theories of basal ganglia function (Alloway et al., 2017; Arber and Costa, 2022).

Here, we sought to confirm previous patterns of corticostriatal and PfN thalamostriatal topography observed in mice and rats in the Mongolian gerbil using AAV-based tracing with quantified analysis of digitized 3D constructions of the fluorescent axonal/terminal labeling. We also used this approach to begin studying vestibular inputs to the thalamus reported in mice. We chose the gerbil to investigate vestibular function due to the easy access to the vestibular apparatus through the overly large bulla, the easy access to the vestibular nuclei through the large cisterna magna, and previous vestibular work in this species (Rennie and Correia, 1994; Newlands et al., 2005; Shinder et al., 2005a,b, 2006; Tinling et al., 2005; Mowery et al., 2023; Wackym et al., 2023; Hong et al., 2024). Our results confirm previous findings in other rodent species and reveal subregional patterns of preferential innervation, including dense motor and somatosensory inputs to the dorsal striatum, and a notable preference for auditory and visual cortices to innervate the ventral portion of the posterior striatum. We also show extensive striatal innervation from the parafascicular (PfN) thalamus throughout the entire striatum, tapering to near absence in the ventral portion of the posterior striatum. Finally, we confirm a pathway in gerbils previously reported in mice by which vestibular information is conveyed to the PfN thalamus via the medial vestibular nucleus (MVN) and then broadcast throughout the striatum. This circuit could provide the real-time vestibular integration for speed and direction to the striatum via PfN thalamostriatal, as described in awake behaving animals (Watson et al., 2021; Fallon et al.*,* 2023). Thus, we provide behavioral evidence for vestibular dysfunction-induced performance deficits in a striatal-mediated 2AFC associative conditioning behavioral paradigm. Taken together, these data indicate homology in patterns of neuroanatomical connectivity across Rodentia and provide a neuroanatomical map to guide future behavioral and physiological vestibular studies in the gerbil and other rodents/species.

## Materials and Methods

### Animals

A total of 40 adult male and female Mongolian gerbils (*Meriones unguiculatus*) met the inclusion criteria and were used in this study (out of 46 total). All animals were housed in the same vivarium facility under a 12 h light/dark cycle with *ad libitum* access to food and water. Stereotaxic injection of anterograde or retrograde AAV was used for anatomical tracing. Opening of the bulla and surgical creation of a 1.5 mm fenestration of the superior (anterior) semicircular canal created the vestibular superior semicircular canal dehiscence (SSCD) resulting in a pathologic third mobile window. Opening of the bulla without creation of a SSCD produced the sham control. The details of this procedure have been published previously (Mowery et al., 2023; Wackym et al., 2023). Exclusion criteria included removal from the study for any animal with persistent circling or head tilt present at 3 d after SSCD (two animals) or if the AAV injection failed or was not in the desired location (four animals). All animal procedures were performed in accordance with the regulations of the Institutional Animal Care and Use Committee.

### AAV injection

Gerbils were anesthetized (isoflurane 2%) and placed in a stereotaxic frame. All stereotaxic locations were derived from coronal plane coordinates in the Mongolian Gerbil atlas (Radtke-Schuller et al., 2016). The left and right temporal bones were exposed. For motor cortex injections, a craniotomy was made at the bregma (ML 1.9 mm; AP 0.45 mm), and the pipette was lowered orthogonally from the pial surface (DV 800 μm). For somatosensory cortex injections, a craniotomy was made at the bregma (ML 4.1 mm; AP −0.95 mm), and the pipette was lowered orthogonally from the pial surface (DV 800 µm). For auditory cortex injections, a craniotomy was made in the temporal bone at the level of core AC bregma (−2.35 bregma), ∼4.5 mm ventral from the edge of the temporal bone, and the pipette was lowered orthogonally from the pial surface (DV 800 µm). For visual cortex injections, a craniotomy was made at the bregma (ML 3.5 mm; AP −4.45; DV 800 μm). For somatosensory striatum retrograde injections, a craniotomy was made at the bregma (ML 4.0 mm; AP −0.95 mm), and the pipette was lowered orthogonally from the pial surface (DV 3.5 mm). For auditory striatum retrograde injections, a craniotomy was made at the bregma (ML 4.8 mm; AP −2.0 mm), and the pipette was lowered orthogonally from the pial surface (DV 3.0 mm). For PfN anterograde injections, a craniotomy was made at the bregma (ML 0.75 mm, AP −2.70 mm), and the pipette was lowered orthogonally from the pial surface (DV 4.5 mm). For each injection, a small durotomy was made over the precise stereotaxic coordinates and a glass pipette (Sutter Instrument) filled with 3 μl of AAV was lowered orthogonally to the pial surface. The pipette was then advanced to the appropriate depth based on target (see above). AAV was injected with a Nanoject III (Drummond) at 10 nl per second until 350 nl was reached. The pipette was left in place for 20 min before being slowly withdrawn.

### AAV vectors

AAV vectors were obtained commercially from Addgene. For anterograde cortical injections, AAV1-CaMKII-eGFP or AAV1-CaMKII-mCherry (7 × 10^12^ vg/ml) was used, with the CaMKII promoter selected to target layer 5 projection neurons, specifically PT and IT types. For retrograde striatal injections, AAVretro-hSyn-eGFP or AAVretro-hSyn-mCherry (7 × 10^12^ vg/ml) was employed, utilizing the hSyn promoter to target all projecting cell types, not limited to cortical pyramidal neurons. For thalamic injections, both anterograde and retrograde applications used AAV1-hSyn-eGFP or AAV1-hSyn-mCherry (7 × 10^12 ^vg/ml) and AAVretro-hSyn-eGFP or AAVretro-hSyn-mCherry (7 × 10^12^ vg/ml), respectively, with the hSyn promoter again chosen to target all projecting cell types. All fluorescent transgenes were allowed to express for ∼3 weeks prior to histological processing.

### Histology

At the end of the experiments, all injected animals were deeply anesthetized with an intraperitoneal injection of Euthasol (300 mg/kg) and perfused with phosphate-buffered saline and 4% paraformaldehyde. Brains were removed, postfixed, and sectioned at 50 µm on a benchtop vibratome (Pelco). All sections (50 µm) were float mounted on pig gel slides (Southern Biotech) and coverslipped with mounting medium (Invitrogen ProLong Antifade with DAPI).

### AAV fluorescence quantification

**Figure 1.**
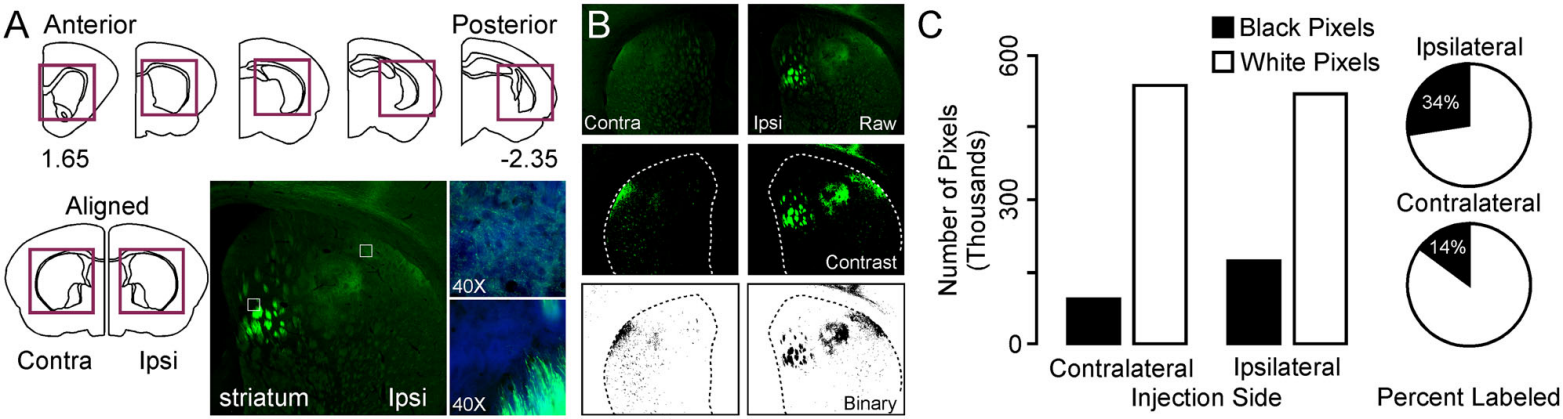
Methodology for the quantification of fluorescent labeling in gerbil brain tissue. ***A***, Diagrams showing coronal sections across the AP axis of the gerbil striatum (top). Red boxes indicate region of interest for quantification. Diagram showing the alignment of contralateral and ipsilateral sections (bottom, left). Photomicrographs of one section in dorsal striatum (bottom, middle) with labeling from synaptic contacts and fibers of passage (bottom, right). ***B***, Photomicrographs showing the raw fluorescent images (top), the contrasted images that eliminate background fluorescence (middle), and the inverted binary (B&W) images that were used for histogram quantification in ImageJ (bottom). ***C***, Bar graph showing the raw histogram of labeled and nonlabeled pixels in striatum (left) and pie chart showing the percentage of labeled pixels (right). Ipsilateral, IPSI; contralateral, Contra.

All images were generated on a Revolve 4 fluorescent imaging system. Figure 1 shows the method used to process the images and reduce them to binary elements for pixel counting. For GFP labeling, exposure time was set to 500 ms. For mCherry labeling, exposure was set to 1,000 ms. LED intensity was set to 100% for both channels. Gain was set at the beginning of each session by referencing a single example for GFP labeling and mCherry labeling (saved primer image). Prior to analysis, imaging of injection sites and associated thalamic nuclei were used to confirm cortical site of interest. For each session, the ipsilateral striatum and contralateral striatum were imaged from the caudal tail of posterior striatum to the rostral dorsal striatum corresponding to the bregma (Fig. 1*A*, top). This approach limited intensity variance between users and sessions. Images were imported into Canvas X (ACD Systems of America). All sections were visually inspected and then arranged within Canvas so that the first section of the posterior ipsilateral and contralateral striatum was aligned. Images were aligned like this through the anterior dorsal striatum (Fig. 1*A*, bottom). Only data from sections between the bregma −2.35 and 1.65 were used in the analyses. After each image was aligned in Canvas with its corresponding ipsilateral and contralateral sections (Fig. 1*B*, top), the striatum was traced with a vector line, and contrast processing was performed to eliminate background fluorescence and reveal AAV fluorescent protein labeling (Fig. 1*B*, middle). This was done with the brightness/contrast function by setting the brightness to ∼35 and the contrast to ∼80. These images were then converted to grayscale and inverted and converted to binary pixels (B&W) corresponding to 255 for white or 0 for black and all shades of gray (Fig. 1*B*, bottom). Images were exported as PNGs and loaded into ImageJ software (NIH). The striatal regions were carefully traced, and a histogram function was performed. The histogram was used to count all black and white pixels. This was exported to excel for statistical processing, where percentage labeling functions were calculated (Fig. 1*C*).

### Auditory brainstem response testing

Animals were anesthetized with isoflurane (1.0%) and placed in a small sound chamber (IAC, Sound Room Solutions). Auditory brainstem response (ABR) recordings were obtained by inserting pin electrodes subcutaneously at the vertex of the skull and just caudal to the right pinna; the ground electrode was inserted at the base of the tail. BioSigRZ software and the TDT ABR system (Tucker-Davis Technologies) were used to collect ABR data. A 10 cm tube (closed field) was inserted into the ear and placed at the opening of the ear canal. The left ear of the animal was stimulated via multifield speaker (MF1, Tucker-Davis Technologies) at 1, 2, 4, 8, and 16 kHz tones [90–20 dB SPL (10 dB steps)], 5 ms, 2 ms linear ramp rise–fall times at 25 Hz. Traces were averaged over 500 (threshold) sweeps. Thresholds for each frequency were measured as the last dB SPL, i.e., 10 dB SPL resolution stimulus level, that elicited a tone-induced ABR.

### Sound-induced cervical positive potential vestibular evoked myogenic potentials

Sound-induced otolithic stimulation and evoked intramuscular excitatory potential recordings were performed by inserting pin electrodes into the neck extensor muscles (splenius capitus *m.*) and the reference electrode in the vertex of the skull [cervical positive vestibular evoked potential (c+VEMP)]. BioSigRZ software and the TDT ABR system were used to collect c+VEMP data. A 10 cm tube capable of delivering 100 dB SPL (see TDT specs, Closed Field) was inserted into the ear and placed at the opening of the ear canal. The left ear of the animal was stimulated via multifield speaker (MF1, Tucker-Davis Technologies) at 2 kHz [100–80 dB SPL (5 dB steps), 5 ms, 2 ms linear ramp rise–fall times sampled at 25 kHz]. Traces were averaged across 500 (threshold) sweeps. The c+VEMPs were recorded under low isoflurane anesthesia (<1.5%), near conditions of wakefulness. The c+VEMP was measured when it appeared under the condition of stimulation of air-conducted sound at 2 kHz and 100 dB. Peak amplitudes were measured by subtracting the peak of the negative N1 wave (in μV) from the later positive P1 wave.

### Auditory discrimination task performance

We assessed auditory decision-making abilities in gerbils using a single-interval alternative forced choice (AFC) task. Briefly, gerbils were given controlled access to food and trained to discriminate between amplitude modulated (AM) broadband noise presented at 4 versus 12 Hz. Gerbils were placed in a behavioral arena test cage housed in a sound-attenuating chamber (Med Associates) and observed via a closed circuit monitor. Auditory stimuli were presented from a calibrated multifield speaker (MF1, Tucker-Davis Technologies) positioned 15 inches above the test cage floor. Sound calibration measurements were made with a 0.25 inch free-field condenser recording microphone (Brüel & Kjær). A modular pellet dispenser (Med Associates, 20 mg) was connected to a trough-type pellet receptacle (Med Associates) placed on the left or right side of the test cage, and a cylindrical nose port with a 1 inch diameter hole (Med Associates) was placed on the opposite side. Sensitive infrared sensors bisected the nose port and pellet receptacles to detect gerbil nose and head entry, respectively. Stimuli, food reward delivery, and behavioral data acquisition were controlled by an iPac computer system running iCon behavioral interfaces (Tucker-Davis Technologies). Gerbils self-initiated trials by placing their nose in the nose port. On each trial, one of two stimulus types was presented. The “left” stimulus consisted of AM broadband noise (25 dB roll-off at 3.5 and 20 kHz; 100% modulation depth) presented at 4 Hz. The “right” stimulus consisted of AM broadband noise presented at 12 Hz. All stimuli were presented at a sound level of 70 dB SPL and were recalibrated daily using a sound level meter. Each session typically lasted 45 min–1 h. Trials were scored as a “correct” when the animal approached the correct side food trough and as “incorrect” when they approached the wrong food trough, depending on whether the left or right stimulus was played. This training continued for 10 d, as all animals were able to achieve above >75% of correct trials by testing day 10. Ten additional days of testing were conducted in animals that received vestibular SSCD (semicircular canal dehiscence) or sham SSCD.

### Superior semicircular canal dehiscence surgery

Animals were anesthetized with isoflurane (1.0%) and prepared for stereotaxic surgery. An incision was made over the nuchal muscles on the left side of the head just posterior to the ear. Once exposed, the nuchal muscles were dissected sharply and then bluntly to expose the left superior bulla. A 5.0 mm opening was made with a 1.5 mm diamond bur. For SSCD surgery, a 2.0 mm fenestration was made down the center of the canal, leaving the vestibular membrane intact. For the SSCD sham surgery, the intact superior (anterior) semicircular canal was directly visualized but was not fenestrated. The open bulla was then sealed/partitioned with Sterile Silastic (Dow Chemical Company) to partition the air-filled bulla from the overlying neck muscles, thereby restoring the normal air-filled middle ear and avoiding a true conductive hearing loss. Condensation on the inner surface of the Silastic seal was an indicator of this restoration of function. Finally, the reattached muscles were secured to the skull with Medbond tissue glue (Stoelting), which allowed c+VEMP testing after the control (sham surgery) procedure. The incision was closed with a running locked 4-0 Vicryl suture (Ethicon US), and topical antibiotic was applied to the wound. Preoperative c+VEMPs and ABRs were performed and repeated on postoperative day 14.

### Statistical analysis

**Figure 2.**
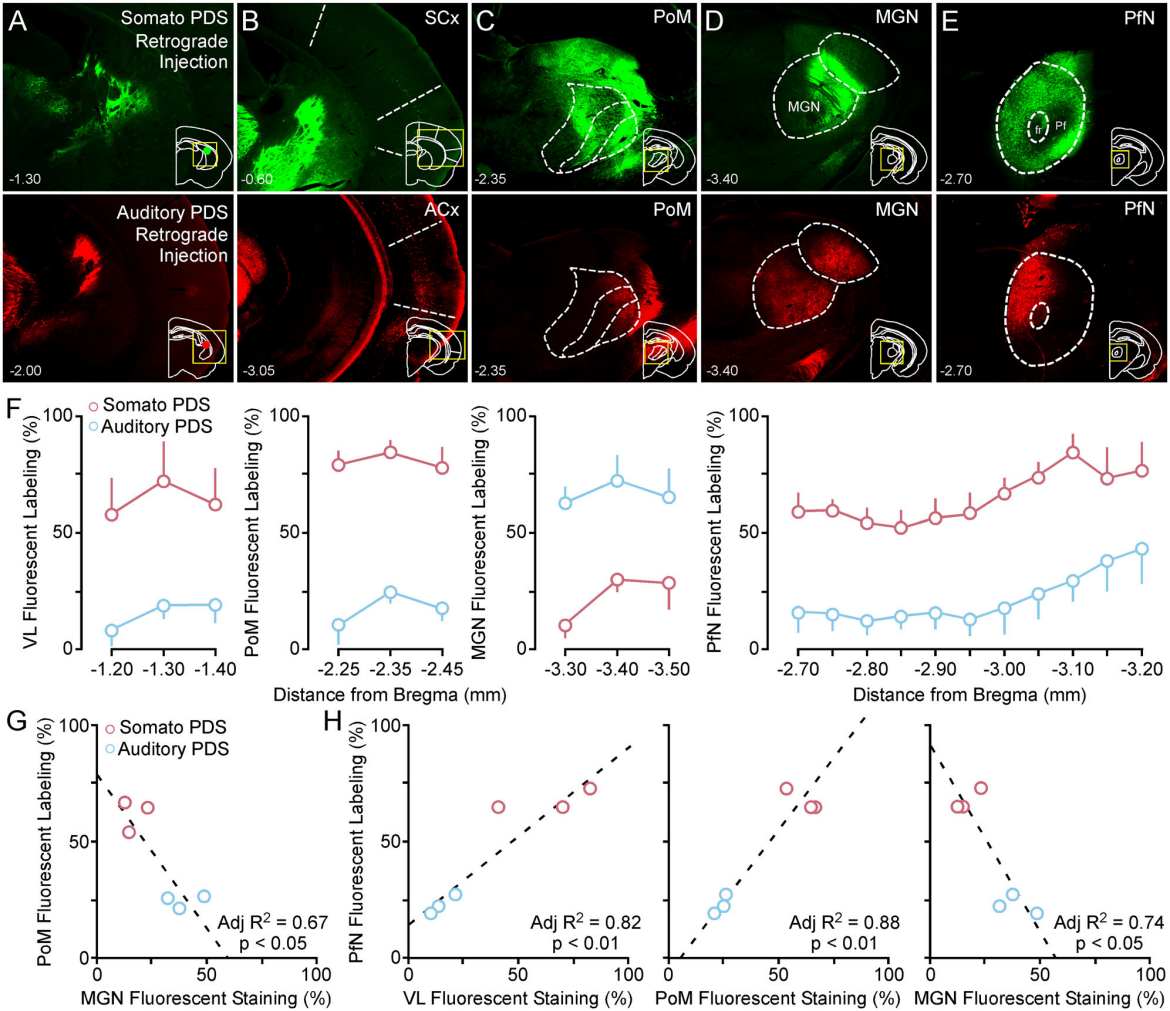
Retrograde labeling of cortex and thalamic nuclei from injection into anterior “somatosensory” and posterior “auditory” regions of posterior striatum. ***A***, Fluorescent micrograph showing retrograde injection site for anterior “somatosensory” PDS (top) and posterior “auditory” PDS (bottom). ***B***, Corresponding cortical labeling of somatosensory cortex (top) and auditory cortex (bottom) L5 pyramidal neurons from anterior “somatosensory” PDS and posterior “auditory” PDS injections. ***C***, Retrogradely labeled somatosensory (PoM) thalamus sections from anterior “somatosensory” PDS (top) and posterior “auditory” PDS (bottom) injections. ***D***, Retrogradely labeled auditory (MGN) thalamus sections from anterior “somatosensory” PDS (top) and posterior “auditory” PDS (bottom) injections. ***E***, Retrogradely labeled PfN thalamus sections from anterior “somatosensory” PDS (top) and posterior “auditory” PDS (bottom) injections. ***F***, Line plots showing significantly higher retrograde labeling for motor (left) and somatosensory thalamus (middle left) from injection of somatosensory PDS compared with significantly higher labeling in auditory thalamus from posterior auditory injection of PDS (middle right). The line plot for the PfN thalamic labeling (right) shows the same trends and motor and somatosensory thalamus with a bias toward the somatosensory striatum injection. For mean difference estimation statistics, see Extended Data Figure 2-1. ***G***, Correlation showing that the somatosensory striatal injection site produces more somatosensory thalamic labeling while the auditory striatal injection site produces more auditory thalamic labeling. ***H***, Correlations showing that the percentage of PfN labeling is positively correlated with motor (left) and somatosensory (middle)thalamic nucleus labeling after somatosensory striatal injection and negatively correlated with auditory (right) thalamic nucleus labeling after auditory striatal injection. Posterior dorsal striatum, PDS; somatosensory, Somato; somatosensory cortex, SCx; auditory cortex, ACx; parafascicular nucleus, PfN; ventrolateral, VL; posterior medial, PoM; medial geniculate nucleus, MGN.

Statistical tests for distribution and significance were performed using the SAS-based package JMP. Due to smaller Ns in some groups (*N* = 3) that lowered statistical power, a conservative statistical approach was utilized (MANOVA with repeated measures) along with estimation statistics, which are useful for datasets with small *n*'s (Ho et al., 2019). For sensorimotor and PfN labeling data comparisons that spanned the A/P axis of the striatum, an ANOVA with a post hoc Tukey’s HSD test was used to compare coordinate matched data and is represented by red-pink-white colored boxes within Figures 4 and 7. For all subregion data presented in the study, measurements were derived from fluorescent staining percentages along anterior-posterior axes and/or mediolateral/dorsoventral planes. Therefore, multiple analyses of variance (MANOVA) utilizing a post hoc repeated measures over matched stereological coordinates were used to compare percent labeling between subregions across animals. Two-group mean difference estimation statistics were used to show mean differences in the raw datasets, as well as to facilitate visualization of the variance between animals. In addition to this, correlations using linear regression with adjusted *R*^2^ analysis were used to show correlative differences based on striatal injection sites (Fig. 2). For behavioral analysis (Fig. 9), a MANOVA with repeated-measures analysis was used to compare behavior between sham and SSCD groups over matched testing days. To compare behavior during specific peaks of vestibular SSCD (postoperative days 6–8 and 13–15), an ANOVA with the post hoc Tukey’s HSD test was used. For behavioral correlations, a linear regression analysis with adjusted *R*^2^ was used.

10.1523/ENEURO.0246-24.2025.f2-1 Figure 2-1 **Estimated Mean Differences for retrograde labeling of thalamic nuclei after injection into somatosensory or auditory striatum. A)** A Gardner-Altman estimation plot showing the mean difference between VL labeling for somatosensory striatum injections versus auditory striatum injections. Both groups are plotted on the left axes; the mean difference is plotted on a floating axes on the right as a bootstrap sampling distribution. The mean difference is depicted as a dot; the 95% confidence interval is indicated by the ends of the vertical error bar; students t-test (t = 6.5), p < 0.001. **B)** A Gardner-Altman estimation plot showing the mean difference between PoM labeling for somatosensory striatum injections versus auditory striatum injections. Conventions are the same as in A; students t-test (t = 11.7), p < 0.001. **C)** A Gardner-Altman estimation plot showing the mean difference between MGN labeling for somatosensory striatum injections versus auditory striatum injections. Conventions are the same as in A; students t-test (t = -4.5), p < 0.01. **D)** A Gardner-Altman estimation plot showing the mean difference between PfN labeling for somatosensory striatum injections versus auditory striatum injections. Conventions are the same as in A; students t-test (t = 15.1), p < 0.001. Download Figure 2-1, TIF file.

## Results

In this study, we used anterograde and retrograde AAVs to neuroanatomically trace the integration pattern of sensory and motor corticostriatal and parafascicular nucleus (PfN) thalamostriatal inputs across the gerbil striatum. First, we used retrograde injection approaches to assess bias in parafascicular innervation of somatosensory and auditory striatal compartments. We then used anterograde corticostriatal (motor, somatosensory, auditory, visual) and thalamostriatal anterograde tracing approaches to explore PfN innervation bias across the A/P striatal axis. Retrograde PfN injections were done to confirm direct input from the medial vestibular nucleus reported in mice. Finally, associative conditioning experiments were carried out to show that vestibular SSCD can impair striatal-mediated behaviors.

### Retrograde neuroanatomical tracing from posterior striatum reveals bias of PfN innervation of somatosensory and auditory striatal compartments

Previous work in rats and mice shows that the striatum has sensory-specific compartmentalization in which certain anterior/posterior stereotaxic coordinates receive greater levels of cortex-specific L5 innervation. Six adult gerbils received retrograde AAV injections into the anterior “somatosensory” (Alloway et al., 2008) compartment (2 males/1 female; Fig. 2*A* top; GFP) or the posterior “auditory” (Znamenskiy and Zador, 2013; Guo et al., 2018) compartment (1 male/2 females; Fig. 2*A* bottom; mCherry) of the posterior striatum. Imaging of the retrogradely labeled inputs showed that the primary cortical inputs were reliably labeled for the “somatosensory” (Fig. 2*B*, top) and “auditory” striatum injections (Fig. 2*B*, bottom). To that end, more somatosensory specific thalamic labeling (%) was seen for the somatosensory injections [e.g., posterior medial (PoM); Fig. 2*C*, top] compared with auditory injections (Fig. 2*C*, bottom). The opposite trend was seen for auditory thalamic labeling (%) with somatosensory injections showing less labeling (%) in medial geniculate nucleus (MGN; Fig. 2*D*, top) compared with auditory injections (Fig. 2*D*, bottom). Finally, there were clear differences in PfN labeling (%) between somatosensory (Fig. 2*E*, top) and auditory striatum injections (Fig. 2*E*, bottom) with a bias toward somatosensory inputs. Figure 2*F* shows that statistical comparison (MANOVA with repeated measures) of the thalamostriatal inputs to the somatosensory and auditory compartments in gerbil [via retrograde labeling (%)] followed the basic trends previously reported in mice and rats (VL Somato PDS vs VL Auditory PDS: *F*_(1,4)_ = 3.70, *p* = 0.018; PoM Somato PDS vs PoM Auditory PDS: *F*_(1,4)_ = 21.50, *p* < 0.001; MGN Somato PDS vs MGN Auditory PDS: *F*_(1,4)_ = 4.46, *p* = 0.013). Here, motor and somatosensory thalamus largely target somatosensory corticostriatal areas of striatum (Smith et al., 2012a,b), and auditory thalamus largely targets corticostriatal auditory areas in the posterior tail of the striatum (Ponvert and Jaramillo, 2019). Retrogradely labeled PfN inputs showed a significantly higher percentage of labeling (%) across the A/P axis of the parafascicular nucleus (bregma; −2.70 to −3.20) for injections made in the anterior “somatosensory” compartment compared with the “auditory” compartment injections (PfN Somato PDS vs Auditory PDS: *F*_(1,4)_ = 27.5, *p* < 0.001). Correlations using linear regression with adjusted *R*^2^ analysis were used to confirm the connectivity pattern between thalamus and striatum in gerbil (Fig. 2*G*,*H*). These analyses were made with matched data from each animal to show the highly conserved labeling (%) patterns associated with somatosensory versus auditory striatum compartment injections. First the difference in somatosensory versus auditory striatum injection sites is made clear by correlating the percentage staining in the somatosensory thalamus (PoM) versus the auditory thalamus (MGN) in Figure 2*G*. This reveals negative correlation between the significantly higher PoM labeling (%) and significantly lower MGN labeling (%) for somatosensory striatum injections and the significantly lower labeling (%) in PoM and significantly higher labeling (%) in MGN for auditory striatum injections (adjusted *R*^2^; PoM = 0.79–1.3 × MGN; *R*^2 ^= 0.67, *p* = 0.028). Correlations between PfN labeling (%) and sensory motor and auditory thalamic labeling (%) based on injections site further reveals a PfN connectivity bias toward the somatosensory striatum in gerbil. There is a very significant positive correlation between the percentage of PfN labeled and motor and somatosensory thalamus (adjusted *R*^2^; PfN = 0.14 + 0.7 × VL; *R*^2 ^= 0.85, *p* = 0.008; Adjusted *R*^2^; PfN = 0.90–1.6 × Somato; *R*^2 ^= 0.79, *p* = 0.016) and a very significant negative correlation between the percentage of PfN labeling (%) and auditory thalamus (adjusted *R*^2^; PfN = −0.03 + 1.14 × MGN; *R*^2 ^= 0.90, *p* = 0.003). Estimation stats were used to test mean differences between the datasets based on injection site (Extended data Fig. 2-1).

#### Patterns of corticostriatal input reveal sensorimotor compartmentalization within the gerbil striatum

**Figure 3.**
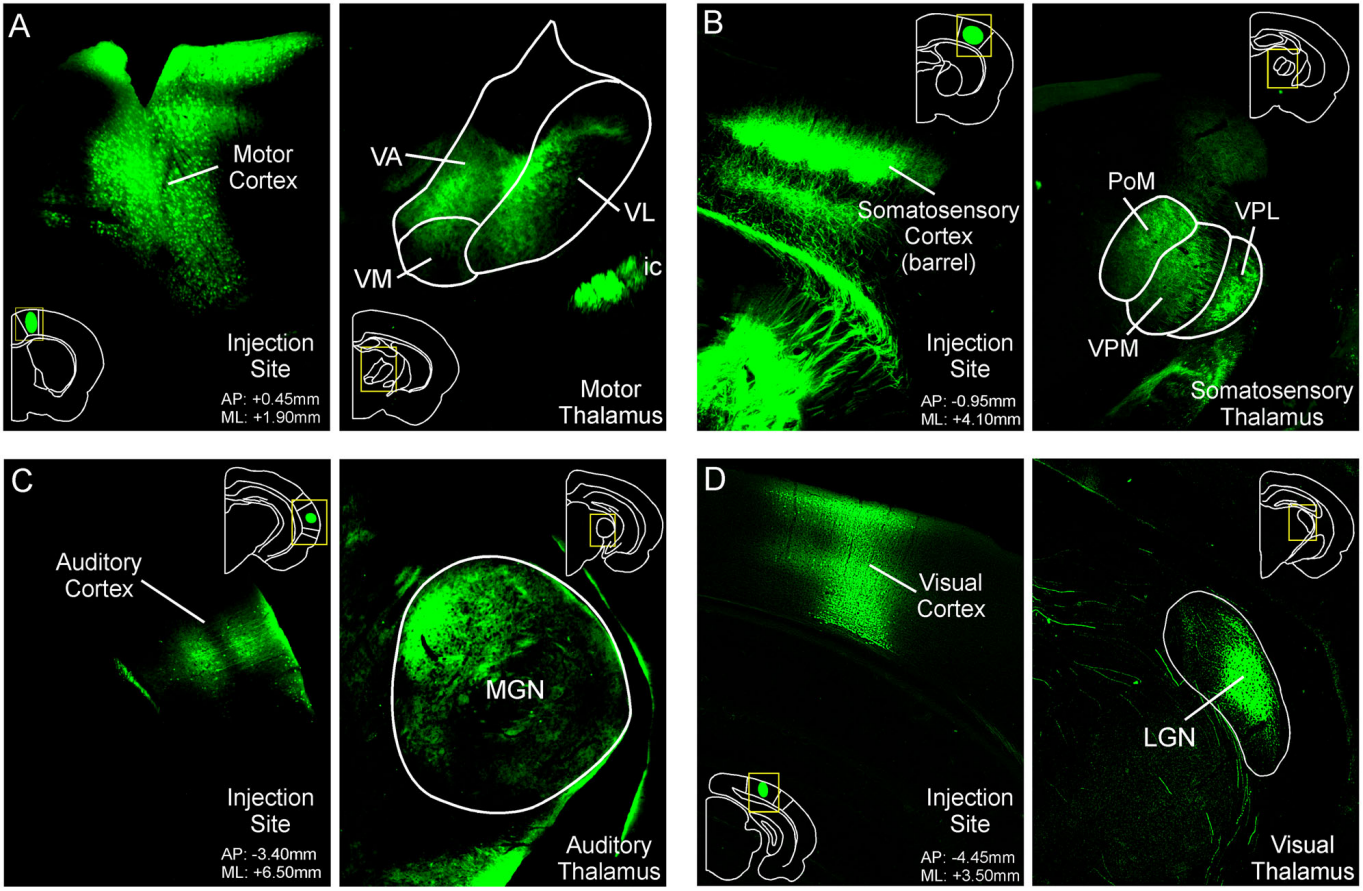
Verification of AAV injection sites used for corticostriatal mapping. ***A***, Fluorescent image of AAV injection into motor cortex (left) and corresponding corticothalamic labeling in the VA, VM, and VL motor thalamus (right). ***B***, Fluorescent image of AAV injection into somatosensory cortex whisker barrel field (left) and corresponding corticothalamic labeling in the PoM, VPM, and VPL of the somatosensory thalamus (right). ***C***, Fluorescent image of AAV injection into auditory cortex (left) and corresponding corticothalamic labeling in the MGN auditory thalamus (right). ***D***, Fluorescent image of AAV injection into visual cortex (left) and corresponding corticothalamic labeling in the LGN visual thalamus (right). Ventral anterior, VA; ventrolateral, VL; ventromedial, VM; posterior medial, PoM; ventral posterior medial, VMP; ventral posterior lateral, VPL; medial geniculate nucleus, MGN; lateral geniculate nucleus, LGN.

**Figure 4.**
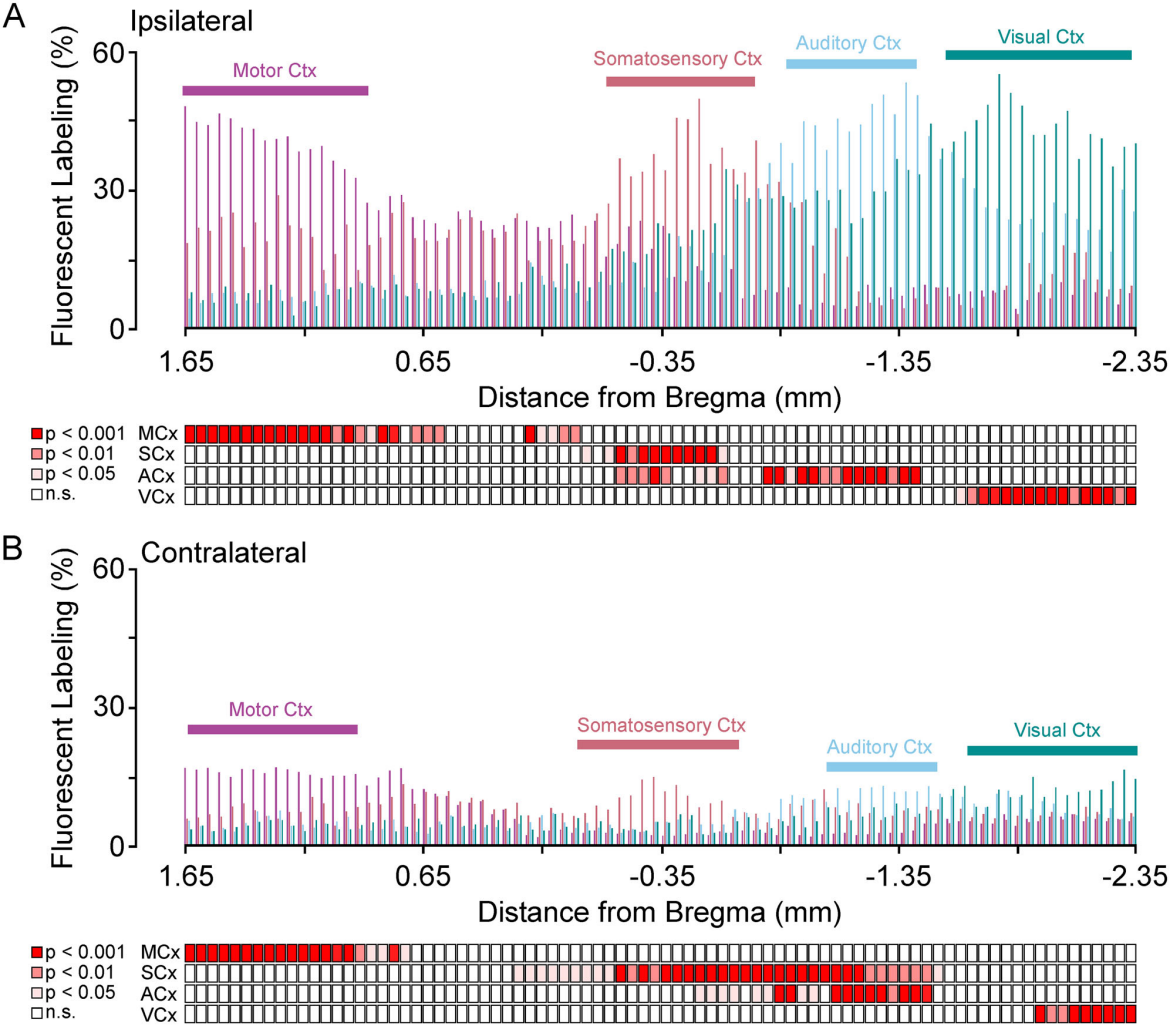
Motor and sensory corticostriatal labeling across the anterior posterior axis of the striatum. ***A***, Bar graphs showing the percentage of ipsilateral corticostriatal labeling for each coronal section across the A/P axis of the striatum for motor, somatosensory, auditory, and visual cortex (top). Corresponding statistical comparisons for significant increases in labeling between motor and somatosensory cortex or auditory and visual cortex reveals compartmentalized peaks of labeling (bottom). ***B***, Bar graphs showing the percentage of contralateral corticostriatal labeling for each coronal section across the A/P axis of the striatum for motor, somatosensory, auditory, and visual cortex (top). Corresponding statistical comparisons for significant increases in labeling between motor and somatosensory cortex or auditory and visual cortex reveals compartmentalized peaks of labeling (bottom). Motor cortex, MCx; somatosensory cortex, SCx; auditory cortex, ACx; visual cortex, VCx*.* Shaded boxes: light pink *p* < 0.05, dark pink *p* < 0.01, red *p* < 0.001, white not significant.

Sensory- and motor-specific compartmentalization has been widely reported in rats and mice (Hoffer and Alloway, 2001; Hoffer et al., 2005; Alloway et al., 2009; Hintiryan et al., 2016; Hunnicutt et al., 2016; Foster et al., 2021). The retrograde tracing data suggested that this is the case for gerbils, so we carried out anterograde corticostriatal tracing experiments in 18 adult gerbils by injecting primary motor (2 males/1 female), somatosensory (2 females/1 male), auditory (4 females/5 male), or visual (1 male/2 females) cortex and then quantified ipsilateral and contralateral labeling (%) from the tail of the striatum to the anterior portion of dorsal striatum (Fig. 3). Male and female animals were divided so that injection sites were based on stereotaxic coordinates from a gerbil atlas (Radtke-Schuller et al., 2016). For each series of injections, imaging of the primary cortex injection site and associated thalamic inputs was used to verify that corticostriatal labeling was from the proper sensory or motor region. Figure 3*A* shows a representative injection site in motor cortex with corresponding labeling in the motor thalamus. Figure 3*B* shows a representative injection site in somatosensory cortex with corresponding labeling in the somatosensory thalamus. Figure 3*C* shows a representative injection site in auditory cortex with corresponding labeling in the auditory thalamus. Figure 3*D* shows a representative injection site in visual cortex with corresponding labeling in the visual thalamus. After verification of each injection site, we then quantified the percentage of ipsilateral or contralateral striatum (relative proportion labeled in percent) across the A/P axis by each cortical input and averaged these across each injection group (Fig. 4). This revealed qualitative peaks of cortical labeling associated with preferential innervation. The Tukey’s HSD test was used to quantify relationships between cortical innervation patterns (Fig. 4*A*, see red shaded boxes below graph). Here motor and somatosensory cortex was directly compared to show A/P positions of significantly more labeling, which signaled compartmentalization in the dorsal striatum. In turn, comparisons between auditory and visual labeling were used to quantitatively reveal peaks associated with compartmentalization in the posterior tail of the striatum. A second feature of this analysis indicated that each cortical area innervated the entire anterior-posterior extent of the striatum (albeit weakly outside its respective hotspot). Finally, each cortical area sent projections across the corpus callosum to the striatum in the contralateral hemisphere, in a homotopic pattern (Fig. 4*B*, see red shaded boxes below graph).

**Figure 5.**
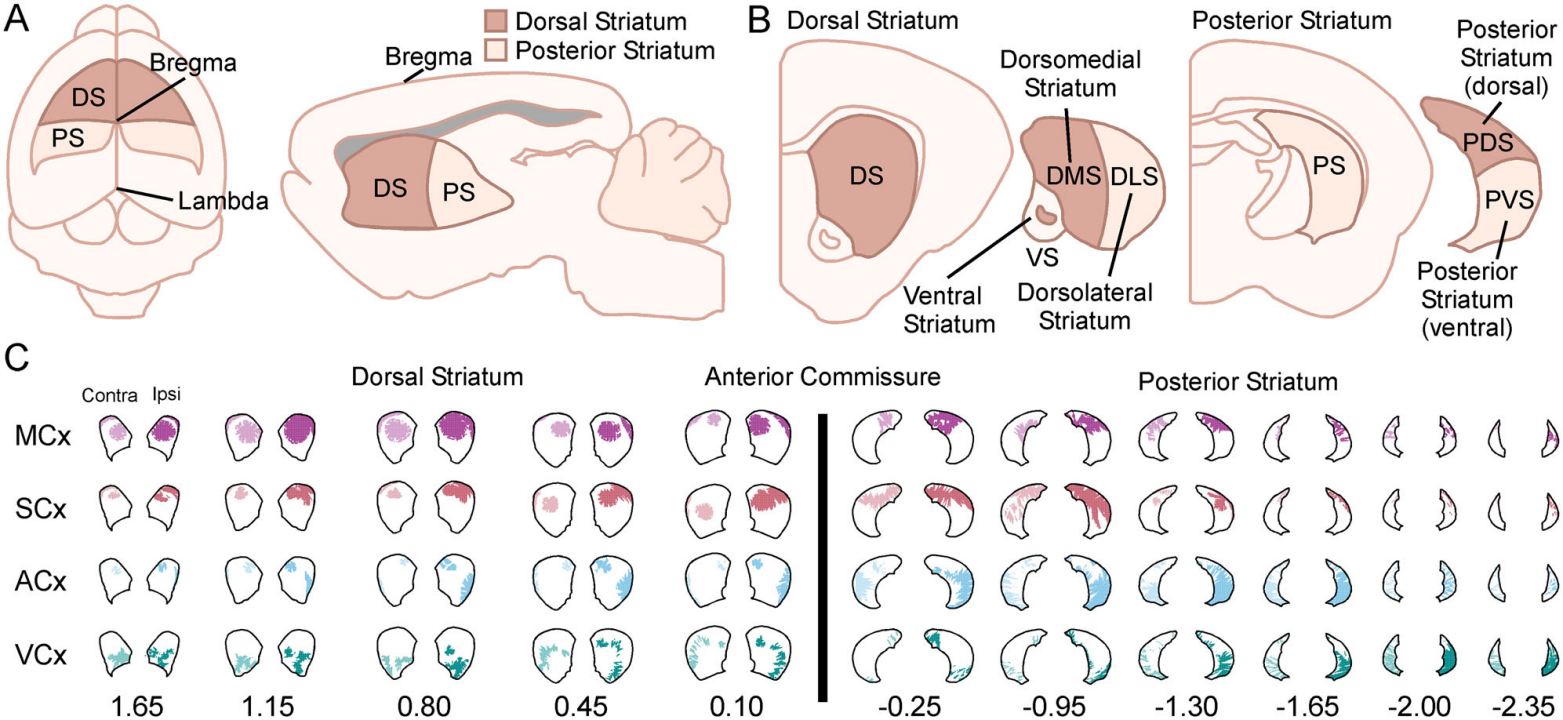
Mediolateral and dorsoventral mapping of dorsal and posterior striatum. ***A***, Top down (left) and Sagittal (right) schematic distinguishing between “dorsal striatum” and “posterior striatum” using the cranial landmark bregma (anterior commissure) as the boundary. ***B***, Coronal schematic showing gerbil striatal subregions of ventral striatum (e.g., nucleus accumbens), dorsomedial striatum (DMS), and dorsolateral striatum (DLS) as well as distinguishing between the dorsal and ventral subregions of posterior striatum (e.g., PDS and PVS). ***C***, Digital reconstructions of the GFP labeled corticostriatal projections from AAV injections into motor cortex, somatosensory cortex, auditory cortex, and visual cortex of gerbil. Reconstructions show the striatum labeling for sections contralateral (left) and ipsilateral (right) to the cortical injection site along the entire anterior posterior extent of the striatum. Ipsilateral, Ipsi; contralateral, Contra; motor cortex, MCx; somatosensory cortex, SCx; auditory cortex, ACx; visual cortex, VCx.

The retrograde labeling from somatosensory and auditory striatum showed sensory modal-specific trends; however, these were not absolutely discrete. This suggested cross-modal representations located within the same dorsoventral plane. Striatal subregions were based on previously published heuristics (Voorn et al., 2004; Hintiryan et al., 2016; Hunnicutt et al., 2016; Hooks et al., 2018; Aoki et al., 2019) to separate common subregions of the striatum for our analysis (Fig. 5). The dorsal and posterior striatum was divided along the A/P axis by the presence of the anterior commissure (Fig. 5*A*). The dorsal and ventral aspects were then divided into the ventral striatum (nucleus accumbens core and shell), dorsomedial striatum (DMS), dorsolateral striatum (DLS) in the dorsal striatum (Fig. 5*B*, left), and dorsal and ventral subdivisions of the posterior striatum (Fig. 5*B*, right, PDS and PVS, respectively). Of the cortical regions injected here, we never observed labeling in the ventral striatum, which contains limbic, olfactory, and gustatory inputs (Fudge et al., 2005; Novejarque et al., 2011) and no analysis was carried out in this region. Qualitative representative examples show clear distinctions of sensory and motor representations in each of these striatal subcompartments (Fig. 5*C*). Visual inspection suggested sensory- and motor-specific dorsoventral and mediolateral compartmentalization, as well as separate areas of cross-modal innervation.

**Figure 6.**
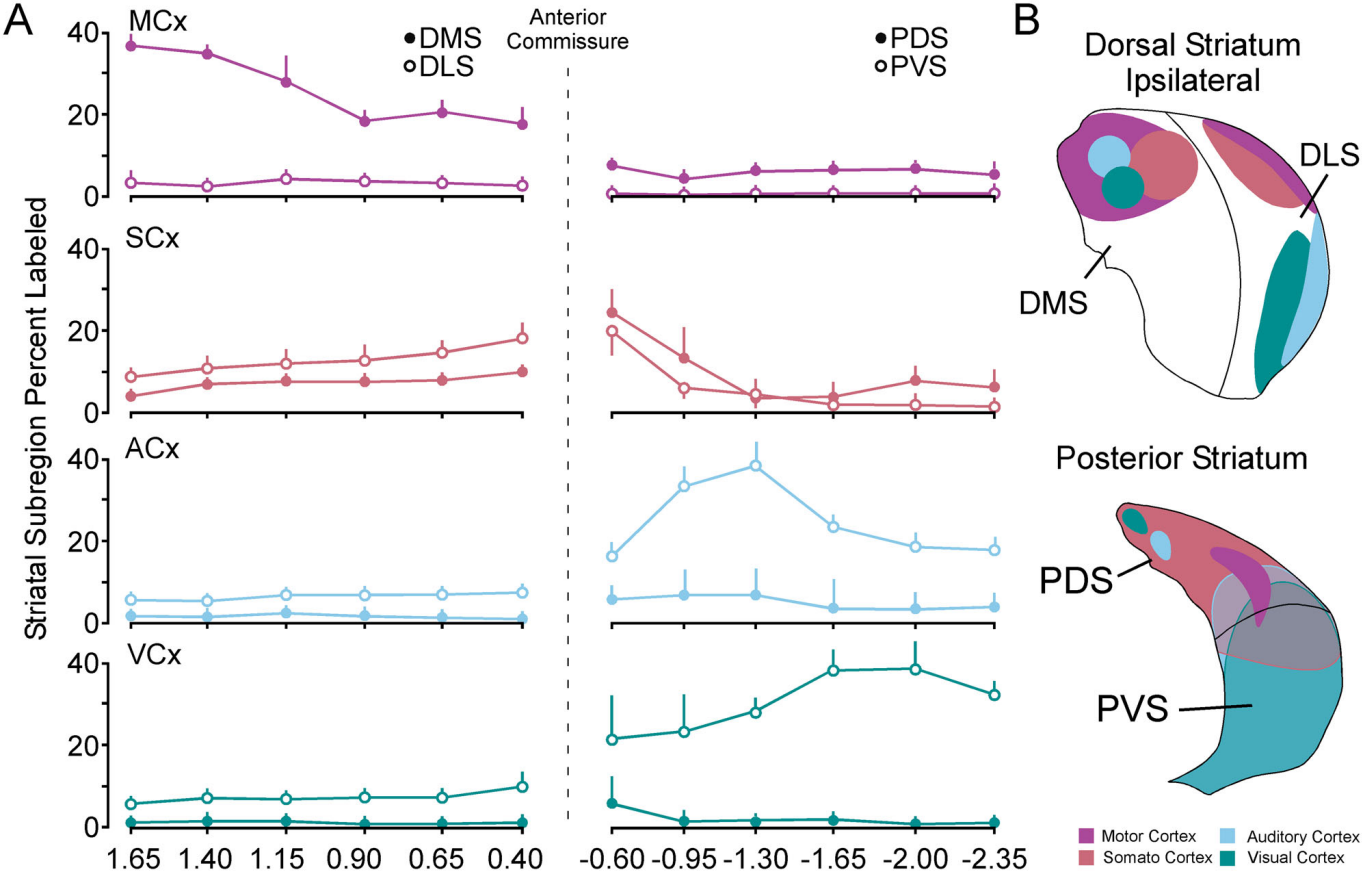
Quantification of mediolateral labeling of the dorsal striatum and dorsoventral labeling of the posterior striatum. ***A***, Line plots showing mediolateral or dorsoventral comparisons of the average labeling at each stereotaxic coordinate over the dorsal (left) and posterior (right) A/P axis of the striatum. Data was separated into dorsomedial and dorsolateral subcompartments in the dorsal striatum and dorsal and ventral subcompartments for the posterior striatum for MCx, SCx, ACx, and VCx corticostriatal labeling. Labeling reveals subcompartment bias as well as compartmentalization (peaks) along the A/P axis for each striatal region. The same trends are seen for contralateral labeling of striatal subcompartments can be seen in Extended Data Figure 6-1. For mean difference estimation statistics for ipsilateral and contralateral labeling, see Extended Data Figure 6-2. ***B***, Diagram showing the general pattern of labeling for motor, somatosensory, auditory, and visual cortex in the dorsal and posterior striatum. Dorsomedial striatum, DMS; dorsolateral striatum, DLS; posterior dorsal striatum, PDS; posterior ventral striatum, PVS; motor cortex, MCx; somatosensory cortex, SCx; auditory cortex, ACx; visual cortex, VCx.

To evaluate these labeling patterns in more detail, we quantified the percentage of GFP labeling in each subregion of the striatum (Fig. 6). The analysis used the arbitrary demarcations (Fig. 5*B*) to quantify the percentage of motor, somatosensory, auditory, and visual labeling in each subregion. Figure 6*A* shows that in the dorsal striatum, comparing medial with lateral subcompartment labeling (%) revealed significantly more labeling in the DMS for motor inputs (MCx DMS vs MCx DLS; *F*_(1,4)_ = 46.9, *p* < 0.001), more balanced labeling (%) between somatosensory inputs with slightly more labeling (%) in DLS (SCx DMS vs SCx DLS; *F*_(1,4)_ = 21.8, *p* < 0.001), and significantly more in DLS for auditory and visual inputs (ACx DMS vs ACx DLS; *F*_(1,4)_ = 17.42, *p* < 0.001; VCx DMS vs VCx DLS; *F*_(1,4)_ = 25.8, *p* < 0.001). For posterior striatum, there was significantly more labeling (%) for motor inputs in the PDS (MCx PDS vs MCx PVS; *F*_(1,4)_ = 21.3, *p* < 0.001), nonsignificant balanced labeling (%) for somatosensory inputs between dorsal and ventral subcompartments (SCx PDS vs SCx PVS; *F*_(1,4)_ = 0.31, *p* = 0.32), and significantly more labeling (%) in the PVS for auditory and visual inputs (ACx PDS vs ACx PVS; *F*_(1,4) _= 24.62, *p* < 0.001; VCx PDS vs VCx PVS; *F*_(1,4)_ = 25.09, *p* < 0.001). Figure 6*B* shows qualitative representations of these dorsoventral-mediolateral analyses in the dorsal (top) and posterior (bottom) striatum. These reveal a distinct pattern of cross-modal and unimodal compartmentalization for these corticostriatal inputs in both the dorsal and posterior striatum of the gerbil. The same statistical trends and pattern of inputs were seen for contralateral labeling (%; Extended Data Fig. 6-1). Estimation statistics were used to compare the mean differences in labeling (%) for striatal subcompartments across the raw data (Extended Data Fig. 6-2).

10.1523/ENEURO.0246-24.2025.f6-1 Figure 6-1 **Quantification of contralateral mediolateral labeling of the dorsal striatum and dorsoventral labeling of the posterior striatum. A**) Line plots showing mediolateral or dorsoventral comparisons of the average labeling at each stereotaxic coordinate over the dorsal (left) and posterior (right) A/P axis of the striatum contralateral to injection sites. Data was separated into dorsomedial and dorsolateral sub compartments in the dorsal striatum and dorsal and ventral sub compartments for the posterior striatum for MCx, SCx, ACx, and VCx corticostriatal labeling. In the contralateral dorsal striatum, comparing medial to lateral sub compartment labeling revealed significantly more labeling in the DMS for motor inputs (MCx DMS vs MCx DLS; F[1,4] = 21.3, p < 0.001), more balanced labeling between somatosensory inputs with slightly more labeling in DLS (SCx DMS vs SCx DLS; F[1,4] = 11.3, p < 0.01), and significantly more labeling in DLS for auditory and visual inputs (ACx DMS vs ACx DLS; F[1,4] = 28.6, p < 0.001; VCx DMS vs VCx DLS; F[1,4] = 9.3, p < 0.05). For posterior striatum, there was significantly more labeling for motor inputs in the PDS (MCx PDS vs MCx PVS; F[1,4] = 8.9, p < 0.01), non-significant balanced labeling for somatosensory inputs between dorsal and ventral sub compartments (SCx PDS vs SCx PVS; F[1,4] = 1.6, p = 0.95), and significantly more labeling in the PVS for auditory and visual inputs (ACx PDS vs ACx PVS; F[1,4] = 35.9, p < 0.001; VCx PDS vs VCx PVS; F[1,4] = 12.5, p < 0.01). Labeling reveals sub compartment bias as well as compartmentalization (peaks) along the A/P axis for each striatal region that correspond to those reported for ipsilateral labeling. **B**) Diagram showing the general pattern of contralateral labeling for motor, somatosensory, auditory, and visual cortex in the dorsal and posterior striatum. *Dorsomedial Striatum, DMS; Dorsolateral striatum, DLS; Posterior Dorsal Striatum, PDS; Posterior Ventral Striatum, PVS; Motor Cortex, MCx; Somatosensory Cortex, SCx; Auditory Cortex, ACx; Visual Cortex, VCx*. Download Figure 6-1, TIF file.

10.1523/ENEURO.0246-24.2025.f6-2 Figure 6-2 **Estimated Mean Differences for MCx, SCx, ACx, and VCx corticostriatal striatum sub compartmental labeling in dorsal and posterior striatum. A)** A Gardner-Altman estimation plot showing the mean difference between MCx labeling for labeling between DMS and DLS in dorsal striatum (left) and PDS and PVS in posterior striatum (right) for ipsilateral (top) and contralateral (bottom) labeled sections. For each estimation plot both groups are plotted on the left axes; the mean difference is plotted on a floating axes on the right as a bootstrap sampling distribution. The mean difference is depicted as a dot; the 95% confidence interval is indicated by the ends of the vertical error bar; MCx DMS vs DLS Ipsilateral students t-test (t = 11.08), p < 0.001; MCx PDS vs PVS Ipsilateral students t-test (t = 12.55), p < 0.001; MCx DMS vs DLS Contralateral students t-test (t = 7.39), p < 0.001; MCx PDS vs PVS Contralateral students t-test (t = 7.63), p < 0.001. **B)** A Gardner-Altman estimation plot showing the mean difference between SCx labeling for labeling between DMS and DLS in dorsal striatum (left) and PDS and PVS in posterior striatum (right) for ipsilateral (top) and contralateral (bottom) labeled sections. Conventions are the same as in A; SCx DMS vs DLS Ipsilateral students t-test (t = -3.79), p < 0.01; SCx PDS vs PVS Ipsilateral students t-test (t = 1.05), p = 0.30; SCx DMS vs DLS Contralateral students t-test (t = -3.08), p < 0.01; SCx PDS vs PVS Contralateral students t-test (t = -0.15), p = 0.88.**C)** A Gardner-Altman estimation plot showing the mean difference between ACx labeling for labeling between DMS and DLS in dorsal striatum (left) and PDS and PVS in posterior striatum (right) for ipsilateral (top) and contralateral (bottom) labeled sections. Conventions are the same as in A; ACx DMS vs DLS Ipsilateral students t-test (t = -10.01), p < 0.001; ACx PDS vs PVS Ipsilateral students t-test (t = -9.16), p < 0.001; ACx DMS vs DLS Contralateral students t-test (t = -11.77), p < 0.001; ACx PDS vs PVS Contralateral students t-test (t = -13.11), p < 0.001.**D)** A Gardner-Altman estimation plot showing the mean difference between VCx labeling for labeling between DMS and DLS in dorsal striatum (left) and PDS and PVS in posterior striatum (right) for ipsilateral (top) and contralateral (bottom) labeled sections. Conventions are the same as in A; VCx DMS vs DLS Ipsilateral students t-test (t = -8.82), p < 0.001; VCx PDS vs PVS Ipsilateral students t-test (t = -11.49), p < 0.001; VCx DMS vs DLS Contralateral students t-test (t = -6.53), p < 0.001; VCx PDS vs PVS Contralateral students t-test (t = -6.85), p < 0.001. *Dorsomedial Striatum, DMS; Dorsolateral striatum, DLS; Posterior Dorsal Striatum, PDS; Posterior Ventral Striatum, PVS; Motor Cortex, MCx; Somatosensory Cortex, SCx; Auditory Cortex, ACx; Visual Cortex, VCx*. Download Figure 6-2, TIF file.

### Patterns of PfN anterograde labeling reveal discrete compartmentalization and sensorimotor innervation bias within the gerbil striatum

**Figure 7.**
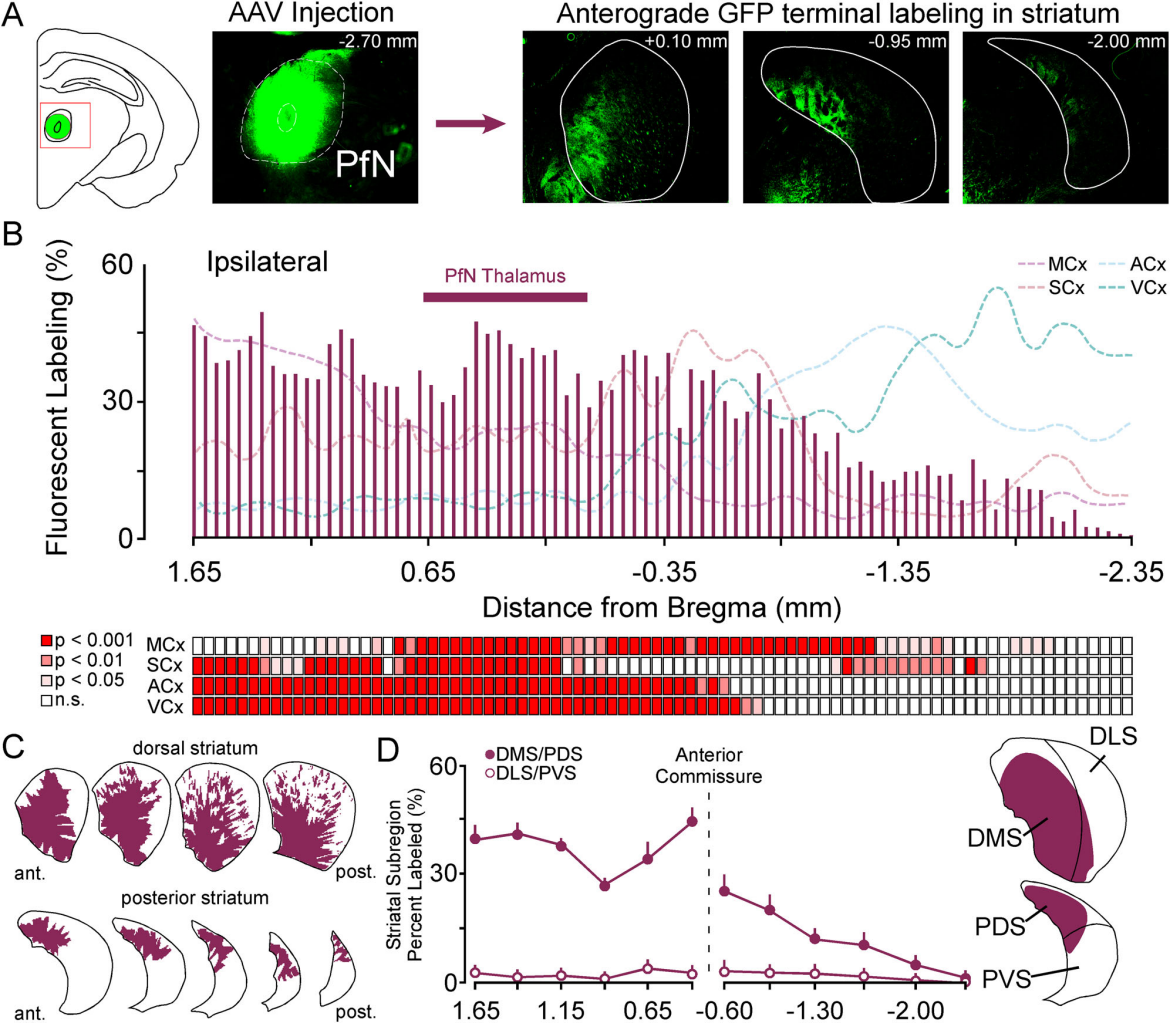
PfN thalamostriatal labeling across the anterior to posterior, mediolateral, and dorsoventral axes of the dorsal and posterior striatum. ***A***, Coronal schematic of the coronal plane of the injection site (left) and Micrographs showing a PfN injection site (middle) and the anterograde labeling in dorsal (+0.10 mm) and posterior (−0.95 & −2.00 mm) striatum. ***B***, Bar graph showing the % labeling of PfN thalamostriatal inputs across the anterior posterior axis of the striatum (top). Dashed colored lines show motor, somatosensory, auditory, and visual labeling from Figure 4. Corresponding statistical comparisons for significant increases in labeling between motor, somatosensory, auditory, and visual cortex reveals compartmentalized peak of labeling (higher than all sensory and motor labeling) between 0.65 and the anterior commissure (bottom). ***C***, Digital reconstructions of the anterograde GFP labeled thalamostriatal projections from PfN. ***D***, Line plots showing the mediolateral or dorsoventral comparisons of the average labeling at each stereotaxic coordinate over the dorsal (left) and posterior (right) A/P axis of the striatum (left). Data was separated into dorsomedial and dorsolateral subcompartments in the dorsal striatum and dorsal and ventral subcompartments for the posterior striatum for PfN thalamostriatal labeling. Labeling reveals subcompartment bias (dorsal and posterior striatum) as well as dorsomedial compartmentalization (peak) in dorsal striatum. Diagram showing the general pattern of labeling for PfN in the dorsal and posterior striatum (right). For mean difference estimation statistics, see Extended Data Figure 7-1. Dorsomedial striatum, DMS; dorsolateral striatum, DLS; posterior dorsal striatum, PDS; posterior ventral striatum, PVS; motor cortex, MCx; somatosensory cortex, SCx; auditory cortex, ACx; visual cortex, VCx. Shaded boxes: light pink *p* < 0.05, dark pink *p* < 0.01, red *p* < 0.001, white not significant.

The retrograde tracing data suggested a somatosensory bias for PfN innervation, so we carried out anterograde PfN tracing experiments in three adult gerbils (2 males/1 female) to map the pattern of labeling across the A/P axis in relation to sensory and motor inputs (Fig. 7). PfN injections (Fig. 7*A*, left) reveal innervation across the entire ipsilateral A/P axis of the striatum (Fig. 7*A*, right). As previously reported (Castle et al., 2005; Wall et al., 2013; Smith et al., 2016), projections to the contralateral striatum were sparse to nonexistent so analysis was not carried out. We quantified this labeling (%) in Figure 7*B* and showed a preferential innervation of the dorsal striatum, tapering off with less dense innervation of the posterior striatum. Interestingly, a discrete region of peak labeling (%) occurs between the peak of motor and somatosensory corticostriatal inputs (Fig. 4*A*) that could indicate a compartmentalized input for PfN (see red shaded boxes below graph). Qualitative reconstructions show that the PfN thalamus has dense ipsilateral innervation of the entire striatum that overlaps with every major subregion (Fig. 7*C*). In Figure 7*D*, quantification of the percentage labeling in the dorsal striatum show a significant bias toward the DMS subcompartment in dorsal striatum (PfN DMS vs PfN DLS; *F*_(1,4) _= 73.3, *p* < 0.001) and PDS subcompartment in the posterior tail of the striatum (PfN PDS vs PfN PVS; *F*_(1,4)_ = 7.64, *p* < 0.01). Both subregions are predominantly innervated by corticostriatal motor and somatosensory inputs (Fig. 6) indicating a significant bias of PfN to innervate striatal regions that receive cortical sensorimotor information (Fig. 7*D*, right). Estimation statistics were used to compare the mean differences in PfN labeling (%) between striatal subcompartments across the raw data (Extended Data Fig. 7-1).

10.1523/ENEURO.0246-24.2025.f7-1 Figure 7-1 **Estimated Mean Differences for PfN thalamostriatal striatum sub compartmental labeling in dorsal and posterior striatum. A)** A Gardner-Altman estimation plot showing the mean difference between PfN labeling for labeling between DMS and DLS in dorsal striatum. For each estimation plot both groups are plotted on the left axes; the mean difference is plotted on a floating axes on the right as a bootstrap sampling distribution. The mean difference is depicted as a dot; the 95% confidence interval is indicated by the ends of the vertical error bar; PfN DMS vs DLS students t-test (t = 19.32), p < 0.001. **B)** A Gardner-Altman estimation plot showing the mean difference between PfN labeling between PDS and PVS in posterior striatum. Conventions are the same as in A; students t-test (t = 4.51), p < 0.001. *Dorsomedial Striatum, DMS; Dorsolateral striatum, DLS; Posterior Dorsal Striatum, PDS; Posterior Ventral Striatum, PfN; Parafascicular Nucleus*. Download Figure 7-1, TIF file.

#### Vestibulothalamostriatal circuit in mouse and gerbil

**Figure 8.**
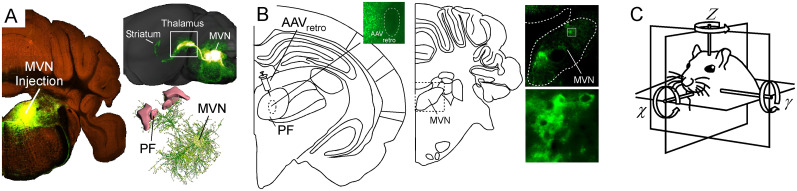
Vestibulothalamostriatal pathway in the mouse and gerbil. ***A***, Photomicrographs from Allen Brain Institute showing labeling from a medial vestibular nucleus (MVN) injection (left) to the PfN thalamus (right) in the mouse. ***B***, Retrograde injection in the PfN thalamus (left) and corresponding labeling in the MVN of the brainstem (right) of the gerbil. ***C***, Diagram showing the encoding of the roll, pitch, and yaw of the head relayed by the medial vestibular nucleus inputs to the PfN thalamus.

There is a vestibulothalamostriatal circuit that relays through the PfN of the thalamus (Fig. 8). The PfN receives direct input from the medial vestibular nucleus in the mouse (Fig. 8*A*; Allen Brain Atlas, https://connectivity.brainmap.org/medial vestibular nucleus). Retrograde tracing of the PfN (Fig. 8*B*) in three adult gerbils (2 males, 1 female) also shows this connection to the medial vestibular nucleus. Vestibular outputs convey information about head tilt, position, and velocity in space relative to the environment (Fig. 8*C*). The PfN has recently been shown to convey this exact type of information to the striatum (Fallon et al., 2023). Thus, this circuit could provide an essential integration site for vestibular information about body position and trajectory with somatosensory proprioceptive inputs and motor output regions of the dorsal and posterior striatum.

#### Asymmetrical vestibular output influences associative conditioning performance

**Figure 9.**
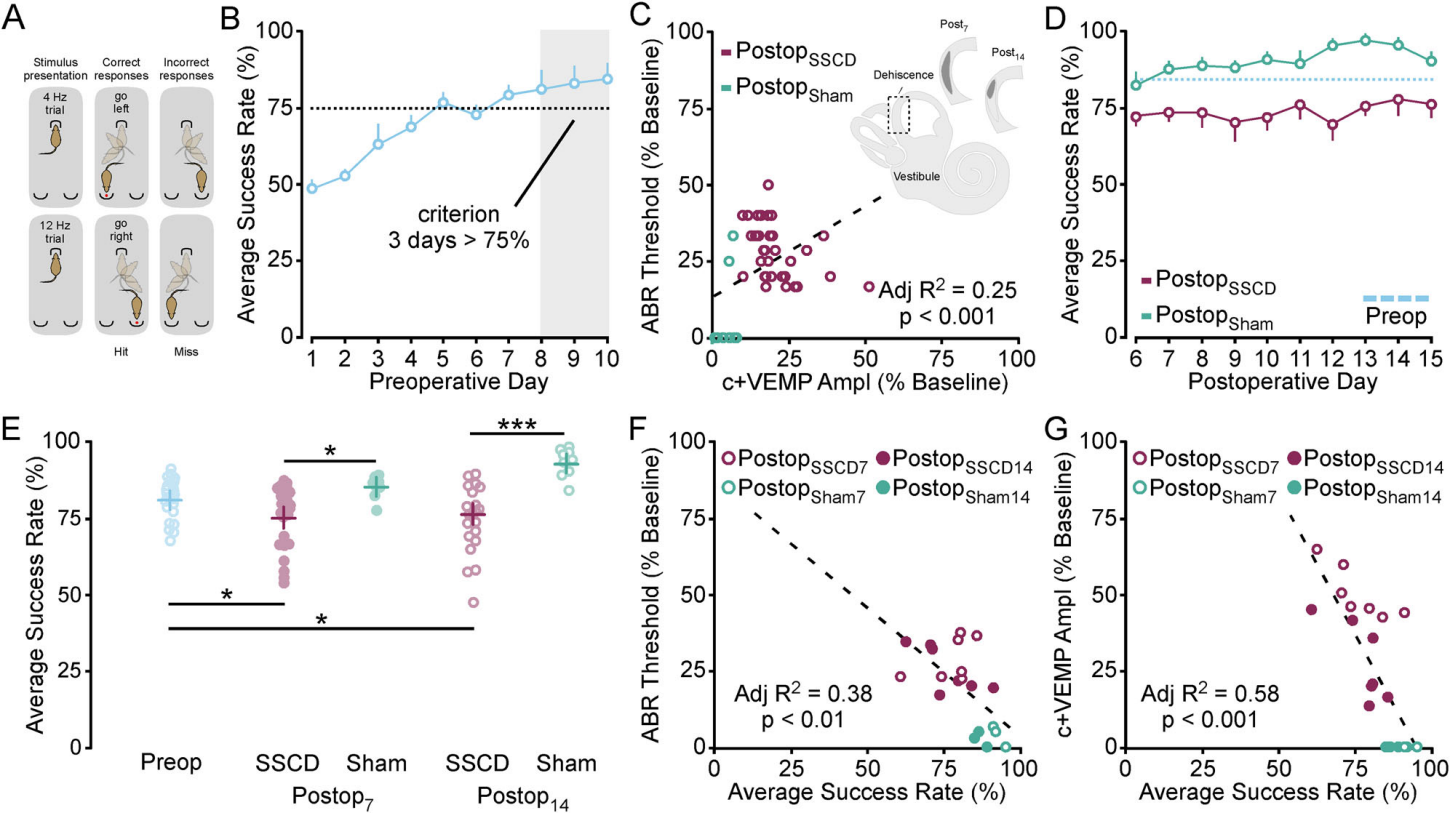
The behavioral effect of asymmetrical vestibular output following the surgical creation of a unilateral superior semicircular canal dehiscence. ***A***, Diagram showing the two-alternative forced choice (2AFC) noise amplitude modulated behavioral paradigm. ***B***, Line graph showing the learning rates for the group of animals and the preoperative scores used to form the baseline for statistical comparisons. ***C***, Correlation showing the relationship between ABR thresholds and cervical positive vestibular evoked myogenic potential (c+VEMP) amplitudes (% difference from baseline) at the end of behavioral testing for SSCD and SSCD sham animals. ***D***, Line plot showing the postoperative performance for the SSCD and sham SSCD groups. Note animals were given 5 d of recovery to avoid acute thigmotaxic behavior. ***E***, Scatter gram showing the comparisons between baseline, sham SSCD, and SSCD animals 7 and 14 d postoperatively. ***F***, Correlation showing the relationship between average success rate (%) and increased ABR thresholds between the sham SSCD and SSCD animals at postoperative day 7 and 14. ***G***, Correlation showing the relationship between average success rate (%) and increased c+VEMP amplitudes between the sham SSCD and SSCD animals at postoperative days 7 and 14. *p < 0.05, **p < 0.01, ***p < 0.001.

The presence of an abnormal third mobile window of the inner ear results in asymmetrical outputs of vestibular information that affects cognitive abilities in humans (Wackym et al., 2016; Wackym et al., 2019). These are associated with impaired memory, learning, and executive function. These patients also experience spatial disorientation including difficulty judging distance, detachment, perceiving the walls or floors moving, and even out-of-body experiences. We have developed an animal model of vestibular dysfunction (Wackym et al., 2023) that induces behavioral decision-making errors in a Go NoGo task (Mowery et al., 2023). For this study we have used this model (5 males, 5 females; adults; >P86) to show that asymmetrical vestibular dysfunction also induces spatial decision-making errors in a left right two-alternative forced choice task (Fig. 9*A*). Here they must discriminate between two modulated noise stimuli that indicate whether a food reward can be retrieved from the left or right food trough. First animals were trained on the task for 10 d to criterion of success rate above 75% (Fig. 9*B*). Animals then received a fenestration of the superior semicircular canal (4 males, 3 females) that induces physiological changes to the cochlear nerve and vestibular nerve outputs or a sham surgery (1 male, 2 females). Figure 9*C* shows that changes in cochlear and vestibular nerve function are significantly correlated with one another (adjusted *R*^2^; ABR threshold = 0.13 + 0.59 * c+VEMP amplitude; *R*^2 ^= 0.25, *p* < 0.001). Dysfunctional vestibular output can be detected by elevations in hearing threshold (ABRs) and sound-induced activation of the otolithic organs that produces amplified neck extensor excitatory potentials of the innervated muscles (c+VEMPs). Both are commonly affected in animal models of vestibular injury (Hirvonen et al., 2001; Carey et al., 2004; Songer and Rosowski, 2007; Attias et al., 2011; Tong et al., 2018; Dlugaiczyk et al., 2019; Curthoys et al., 2023; Wackym et al., 2023) and humans with similar disorders (Wackym et al., 2016, 2019; Bi et al., 2017) and are indirect measures of the magnitude of vestibular dysfunction and its associated symptomology (e.g., nystagmus, imbalance, cognitive impairment). Figure 9*D* shows that the SSCD animals performed significantly worse than the SSCD sham animals over 10 d of post-fenestration testing (MANOVA: *F*_(1,8)_ = 5.693, *p* = 0.032). Figure 9*E* shows that compared with baseline performance, SSCD resulted in a significant decrease in the percentage of correct trials 1 week and 2 weeks after SSCD [mean success rate (%) ± SEM: preop 83.5 ± 1.4 vs postop_7_ 78.0 ± 1.6; *q* = 2.00, *p* = 0.015; preop 83.5 ± 1.4 vs postop_14_ 79.2 ± 1.6; *q* = 2.00, *p* = 0.034]. The percentage of correct trials for the SSCD group was also significantly different from the sham SSCD group 1 week and 2 weeks after SSCD [mean success rate (%) ± SEM: sham injury_7_ 86.0 ± 2.8 vs postop_7_ 78.0 ± 1.6; *q* = 2.00, *p* = 0.022; sham injury_14_ 92.5 ± 3.1 vs postop_14_ 79.2 ± 1.6; *q* = 2.03, *p* < 0.001]. In fact, the SSCD prevented behavioral improvement as evidenced by the better performance of the sham SSCD groups with 2 additional weeks of training [mean success rate (%) ± SEM: sham injury_14_ 92.5 ± 3.1 vs preop 83.5 ± 1.4; *q* = 2.41, *p* < 0.001]. Behavioral performance errors were highly correlated with vestibular (Fig. 9*F*) and auditory (Fig. 9*G*) function, where better performance was significantly correlated with lower ABR thresholds (adjusted *R*^2^; ABR threshold = 0.80–0.73 * % correct; *R*^2 ^= 0.38, *p* < 0.01) and highly significantly correlated with normal vestibular output (adjusted *R*^2^; c+VEMP amplitude = 0.62–0.59 * % correct; *R*^2 ^= 0.58, *p* < 0.001).

## Discussion

**Figure 10.**
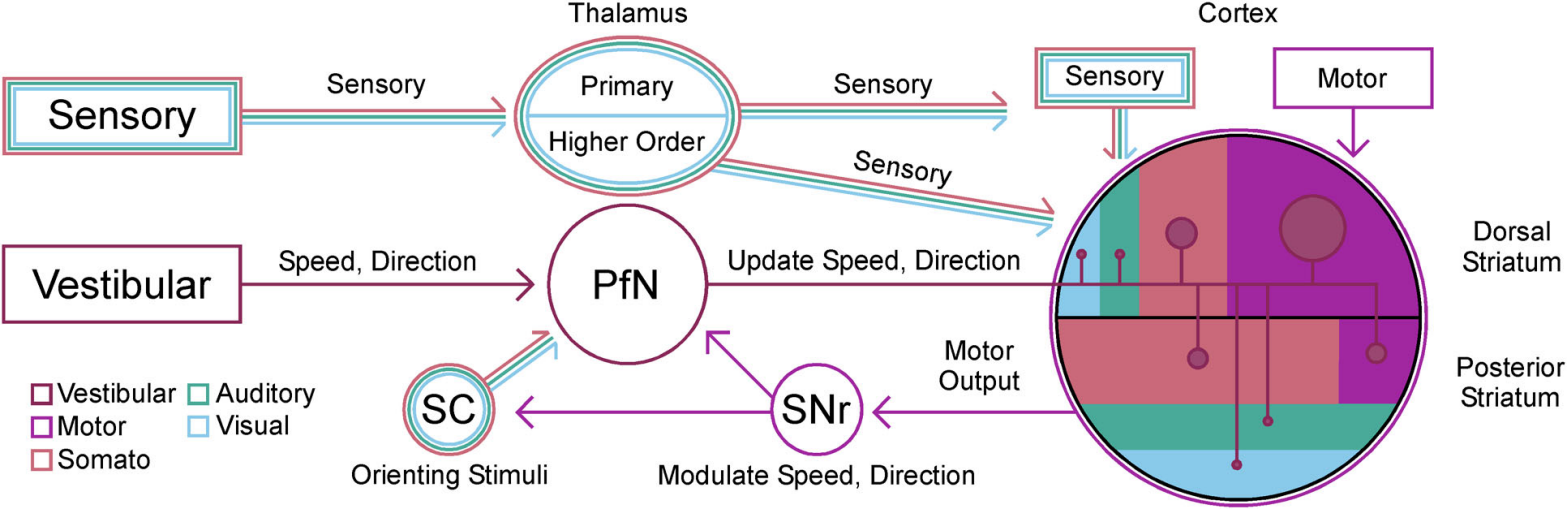
Circuit diagram showing the relay of vestibular information to the entire striatum via PfN inputs. Sensory information is conveyed through the primary thalamic nuclei to sensory cortex, and to higher order thalamus (e.g. PoM, pulvinar), which innervate sensory cortex and striatum directly. Vestibular information is relayed through the parafascicular nucleus of the thalamus (PfN) to all of striatum. The striatum is shown divided by dorsal and posterior striatum, with color-coded regions to represent the general percentage of inputs from each cortical area described in this study. Relative percentages of PfN inputs are represented by circle size. The superior colliculus (SC) and substantia nigra reticulata (SNr) are situated within the PfN circuit to modulate vestibular information about speed and direction via incoming orienting stimuli and striatal motor output.

In this comparative neuroanatomy study, we used the Mongolian gerbil (*M. unguiculatus*), a less widely explored animal model, to systematically measure the patterns of sensory and motor corticostriatal innervation in the dorsal and posterior striatum as they relate to PfN thalamostriatal input. Retrograde tracing experiments suggested the presence of sensory compartmentalization that was verified by cortical anterograde tracing experiments. Anterograde tracing experiments from PfN thalamus revealed a significant bias of thalamostriatal innervation toward regions of striatum receiving somatosensory and motor cortical inputs; namely, in the dorsal medial and posterior dorsal subcompartments of the striatum. Based on these anatomical confirmations, the second part of this study began to assess the functional relevance of these vestibular thalamostriatal inputs. Retrograde tracing of PfN thalamus confirmed direct inputs from the medial vestibular nucleus of the brainstem for a circuit reported in mice. A vestibular superior semicircular canal dehiscence (SSCD) model was then used to show that asymmetrical vestibular dysfunction can impair a striatal-mediated associative conditioning task. Together, the results of this anatomical study suggested that a vestibular striatal circuit exists, which would provide important information about animal speed and direction as it navigates the environment (Fig. 10). The PfN inputs to the striatal compartment are heavily biased toward the sensorimotor inputs of the dorsomedial striatum. If these convey vestibular information, it would provide a functional pathway to integrate motor patterns and joint position with the general vector of the animal's head in space as it locomotes toward a goal. We find bilateral patterns of innervation throughout the brain; however, the PfN innervation of striatum is almost entirely unilateral. Unilateral activation or suppression of this pathway would provide the ability to gradually or abruptly change direction left and right or even turn completely. We see evidence of this with the acute thigmotaxic behavior associated with unilateral vestibular SSCD and other types of vestibular injury. Recent work by Fallon et al. (2023) established kinematic processing within the PfN, and future experiments should manipulate the superior colliculus, substantia nigra pars reticulata, and vestibular functional connectivity to the PfN to assess influence on animal speed and direction. Furthermore, it will be important to understand PfN contributions relative to other thalamostriatal projections. For example, we have previously demonstrated opposing effects of tecto-incertal-thalamic effects in the pulvinar/PoM versus PfN on striatal function (Alloway et al., 2014, 2017; Watson et al., 2015; Watson and Alloway, 2018). The addition of manipulations to vestibular inputs along these pathways will offer vital insight into vestibular modulation of striatal-mediated behavioral output and allow the refinement of current models.

### Comparative neuroanatomical innervation of the striatum in gerbil to other species

Comparative neuroanatomy is very useful for interpreting functional electrophysiological and behavioral studies across animal species, especially as they might relate to human disease and disorders. Our results are consistent with similar studies in other rodents, indicating homology in gross neuroanatomical connectivity patterns within the cortico-basal ganglia circuit across Rodentia. As in previous reports, we found compartmentalized (e.g., patch/matrix-like, see Graybiel, 1990) regions of striatal cells innervated by a primary sensory or motor system, with other regions receiving overlapping sensory and motor inputs (Graybiel, 1984; Donoghue and Herkenham, 1986; Kincaid and Wilson, 1996; Voorn et al., 2004; Hintiryan et al., 2016; Hooks et al., 2018; Aoki et al., 2019; Smith et al., 2022). This type of labeling has also been reported for cortical, thalamic, and subcortical striatal inputs in the nonhuman primate (Selemon and Goldman-Rakic, 1985; Sadikot et al., 1992a,b; Haber et al., 2000; McFarland and Haber, 2000; Borra et al., 2021) and cat (Ragsdale and Graybiel, 1988; Smith et al., 2022), suggesting a common pattern across many species. Our results show roughly equivalent labeling of striatum from each injected cortical region, with differentially distributed GFP expression across striatal subregions. Specifically, motor cortex primarily targeted DMS and some DLS. Auditory and visual cortices primarily targeted the ventral portion of the posterior striatum (PVS). Somatosensory cortex projections were evenly distributed across all subregions but highest in the DLS. In addition to the dense cortical targeting of specific subregions, we observed a diffuse, weak projection to all striatal subregions. This “each-to-all” connectivity paradigm has important implications for how we conceive of striatal subregional function, suggesting a broader, global integration of all cortical information by the striatum, with some areal specialization defined by “hotspots” of hyper-dense innervation from specific cortical areas (e.g., audio-visual in PVS).

Corticostriatal projections to the ipsilateral striatum were dominant, with contralateral projections representing approximately one-third of the percentage labeling of innervation in comparison, consistent with data from other species (Wilson, 1986; Wright et al., 1999, 2001; McHaffie et al., 2001; Alloway et al., 2006; Reig and Silberberg, 2016; Borra et al., 2021; Rico et al., 2024). These bilateral projections arise predominantly from the layer 5 cortical IT and PT projection neurons (Wilson, 1987; Reiner et al., 2010; Shepherd, 2013). The functional purpose of these distinct subtypes of cortical projection neurons are theorized to be motor planning (IT type) versus motor action (PT type). The finding that the ipsi-to-contra-corticostriatal ratio is the same across cortical areas and species indicates a common principle in the evolution of IT-type and PT-type corticostriatal neurons (Wilson, 1987; Reiner et al., 2003, 2010; Alloway et al., 2006) despite some divergence of these types of projection neurons (Gămănuţ et al., 2018). The functional purpose of these distinct subtypes of cortical projection neurons are theorized to provide excitatory drive to the direct and indirect pathway (Lei et al., 2004; Castle et al., 2005; Deng et al., 2006).

Thalamostriatal parafascicular nucleus inputs have been reported in rodents (Dubé et al., 1988; Berendse and Groenewegen, 1990; Lanciego et al., 2004; Castle et al., 2005; Alloway et al., 2014; Gonzalo-Martín et al., 2024) non-human primates (Smith and Parent, 1986; Sadikot et al., 1992a,b; Smith et al., 2004), and cats (Beckstead, 1984; Ragsdale and Graybiel, 1991). The pattern of labeling of gerbil PfN thalamostriatal inputs follows the previously established heuristics that report vast labeling of the dorsal and ventral striatum, with specific density in the motor DMS regions. Our findings in gerbils shows a bias toward motor and somatosensory regions and away from auditory and visual regions, which were reported previously in rats (Alloway et al., 2017) and mice (Voorn et al., 2004; Hunnicutt et al., 2016; Hooks et al., 2018; Aoki et al., 2019); however, we do see some PfN retrograde labeling posterior striatum injections associated with auditory and visual striatum as reported previously too (Mandelbaum et al., 2019; Foster et al., 2021). Identifying other thalamic sources of modulatory and driving influence of the medium spiny neurons located in auditory and visual striatum will provide important nuance to the greater theoretical framework of direct and indirect pathway function. So far studies have reported that auditory thalamic inputs through the MGN to the posterior tail of the striatum exert significant influence on striatally mediated behavior and medium spiny cell function (Znamenskiy and Zador, 2013; Chen et al., 2019; Ponvert and Jaramillo, 2019); however, visual information is not relayed through a thalamostriatal LGN input (Hunnicutt et al., 2016). We do see retrograde labeling of LGN after our striatal injections into the posterior striatum; however, these are likely derived from AAV transfection of the dense bundles of fibers of passage located in this region. One candidate is the lateral posterior nucleus (Juavinett et al., 2020; Scholl et al., 2021; Leow et al., 2022), which is the pulvinar in nonhuman primates (Adams et al., 2000) and cats (de Souza et al., 2021). LP receives visual input and has thalamostriatal output to the posterior tail of the striatum in rodents (Takada, 1992; Funaki et al., 1998; Kobayashi et al., 2007).

### Is there a compartmentalized vestibular representation in striatum?

The presence of vestibular evoked activity in the striatum was demonstrated decades ago (Potegal et al., 1971; Matsunami and Cohen, 1975; Liedgren and Schwarz, 1976). In these early experiments, electrical stimulation of the vestibular nucleus was found to elicit short latency neural responses in the caudate nucleus of anesthetized monkeys, but it is not clear whether this was monosynaptic, disynaptic, or multisynpatic activation. Interestingly, activity was seen in the dorsomedial aspects of the caudate, which aligns with the same location we observe the most intense PfN labeling, the dorsomedial striatum (DMS). In our tracing experiments, we do not observe direct projections from MVN to striatum but do confirm projections from MVN to PfN thalamus which in turn vastly innervates the entire striatum (Smith et al., 2014). These observations led us to hypothesize that the PfN distributes vestibular information widely to caudate/putamen, encompassing each of the dorsal and posterior striatal subregions, but notably the DMS. Further work is needed to more precisely map the axonal innervation pattern of MVN to PfN, importantly to assess whether all neurons in PfN receive vestibular inputs or only a subset. This can then be compared with the known topography of Pf thalamostriatal projections (Mandelbaum et al., 2019), and one can infer the extent of distribution of vestibular signals to striatum from PfN. Importantly, PfN also projects to every other basal ganglia nucleus, including GPe, GPi, STN, SNr, and SNc, suggesting the vestibular signal in PfN may be more widely broadcast to the entire basal ganglia.

Vestibular output is divided into information related to different features of peripherally encoded states corresponding to the angular orientation and momentum of the head in space through four subnuclei (Hitier et al., 2014). The superior, lateral, medial, and inferior vestibular nuclei send out many projections to the oculomotor nuclei, spinal cord, cerebellum, thalamus, and short-range connections to the contralateral nucleus (Straka et al., 2005; Highstein and Holstein, 2006). The medial vestibular nucleus is the largest and receives most of its input from the cristae ampullares of the semicircular canals, which code for the position of the animal's head in space and its directional speed. It forms a two-synapse relay to the striatum through the parafascicular nucleus of the thalamus (Shiroyama et al., 1999; Lai et al., 2000; Watson et al., 2021), which provides the striatum with continuously updated information about direction and speed of the head for body orientation and motor steering (Fallon et al., 2023). Basic vestibular nucleus lesion studies (Shima, 1984; Ishiguro et al., 2007), genetic vestibular dysfunction studies (Kaiser et al., 2001), and surgical creation of an SSCD (Wackym et al., 2023) show a straightforward effect of asymmetrical vestibular dysfunction and behavior in the form of acute thigmotaxic circling. Beyond this, we have shown behavioral impairments to basic features such as balance and higher order cognitive and spatial associative conditioning behaviors that are impaired while the vestibular dysfunction is present (Fig. 9; Mowery et al., 2023; Hong et al., 2024). Together there is ample evidence to support the concept that the vestibular system is influencing striatal activity.

It is intuitive that given the compartmentalization of motor, sensory, olfactory, and other nonsensory systems within the striatum (limbic-emotional, frontal-motor, insular-gustatory, etc.) that a region devoted to vestibular processing should also exist. The pattern of innervation that gives rise to these compartments typically involves a sensory-specific corticostriatal and/or thalamostriatal input densely innervating a specific region of the striatum and a sparser diffuse set of inputs to adjacent striatal territories that create cross-modal integration sites (Reig and Silberberg, 2014). Here we have identified a vestibular PfN thalamic relay that densely innervates a region of the striatum between 0.65 bregma and just in front of the anterior commissure in Gerbil. We also report diffuse weaker overlapping inputs throughout the rest of the striatum in the same conventions associated with motor, somatosensory, auditory, and visual corticostriatal innervation. If there is vestibular striatum, we could expect this region to be innervated by layer 5 cortical inputs from a cortical region approximate to a “vestibular” cortex. Vestibular cortex has been reported in multiple species of monkey's, cats, and humans (Ventre-Dominey, 2014), and widespread activation of various cortical areas after vestibular pathway stimulation has been described in the rodent (Rancz et al., 2015). In primates, cats, and humans, the functional cortical activation is largely connected to head yaw, pitch, and roll, which is vestibular information. Structural and functional mapping in the human has identified at least five distinct pathways between the vestibular nuclei, thalamus, and parietal cortex that can be explored in high-throughput rodent tracing studies. Importantly, PfN itself does not receive input from any sensory cortices, instead having a topographically organized reciprocal innervation with frontal cortex (mPFC to medial PfN, secondary motor cortex to lateral PfN) that is primarily focused on the deep layers 5 and 6 (both input and output for M2-PfN). Interestingly, in rats the M1 projections to PfN are weak (Alloway et al., 2008) compared with M2 cortex, an area that primarily targets DMS as well. So cingulate and/or M2 cortex in rodents seem like good candidates for primary representations of “vestibular cortex,” though it could be broadly distributed from M2 to many parts of cortex (Hooks et al., 2018). At the same time, the studies in cats, nonhuman primates, and humans indicate regions of vestibulothalamic input to various regions across the cortex (especially parietal-insular vestibular cortex and its homologs). Therefore, we hypothesize that there is not a singular vestibulothalamo recipient cortical region for vestibular processing (likely many), but the results from this study do provide evidence for such a region in the basal ganglia through the ascending MVN to PfN thalamostriatal system that innervates a compartment of the DMS. Further work will have to investigate striatal labeling from other vestibulothalamic inputs to the dorsomedial and other compartments of the striatum.

### Limitations of the model and study design

The neuroanatomical results presented here build on our knowledge of the connectivity of the cortico-basal ganglia system across mammalian species and suggest these pathways are present in the Mongolian gerbil. This animal has a strong scientific foundation for auditory (Mowery et al., 2017; Paraouty and Mowery, 2021) and vestibular research (Rennie and Correia, 1994; Newlands et al., 2005; Shinder et al., 2005a,b, 2006; Tinling et al., 2005; Mowery et al., 2023) and great visual acuity (Baker and Emerson, 1983; Wilkinson, 1984; Jacobs and Deegan, 1994; Yang et al., 2015) and is highly trainable and especially suited for awake behaving perceptual and cognitive electrophysiological experiments (Caras and Sanes, 2015, 2017; Ihlefeld et al., 2016; Green et al., 2017; Yao and Sanes, 2018, 2021; Paraouty et al., 2020, 2023; Anbuhl et al., 2022; Yao et al., 2023; Macedo-Lima et al., 2024). The peripheral vestibular system is highly accessible through the overly large bulla that these animals possess (Wackym et al., 2023), they have a large cisterna magna which allows easy access to the vestibular nuclei for tracing/recording, and they are well suited for experimental designs involving vestibular and cochlear manipulation in the periphery (Chamberlain, 1977). Thus, the gerbil is a valuable research tool for investigating the vestibular striatal circuits in rodents.

At the same time, this model suffers primarily from the absence of a foundational body of somatosensory and motor research. No official genetically or neurochemically derived striatal subcompartmentalization for dorsal or posterior striatum has been reported for the gerbil which was a limitation of our analysis. We instead relied upon an approximate division of striatal subregions based on previously published heuristics (Voorn et al., 2004; Hintiryan et al., 2016; Hunnicutt et al., 2016; Hooks et al., 2018; Aoki et al., 2019) to separate common subregions of the striatum for our analysis (Fig. 5). Furthermore, certain genetic and other tools available in the mouse have not been established for gerbils preventing certain types of experimental designs that allow investigations of the patch/matrix compartments (e.g., mu-opiod receptors; Crittenden and Graybiel, 2011). In addition to this, cre lines and genetic lines that allow study of the striatal receptors (D1 vs D2), neuronal subtypes (e.g., exo-patch; Smith et al., 2016), and pathways (direct/indirect) will require the use of cell type specific AAVs (e.g., Allen Brain Institute; Hunker et al., 2024).

Finally, it is important to note that the results presented here do suffer from lower statistical power. For many of these tracing experiments, we only used three animals. Here we have included estimation analyses (Extended Data Figs. 2-1, 6-2, 7-1) which are useful for making statistical interpretations involved experiments with low power due to small numbers of subjects. In some cases, smaller groups are ideal for investigating new phenomena. Here, power analyses are useful for determining the minimum number of animals required to attain reliable significance. This allows for two stage approaches to experimentation with an exploratory phase followed by further confirmation studies. The current study is an example of an exploratory study. Further experiments will revisit these gross anatomical findings in this and other rodent models to more precisely define the functional connectivity between the vestibular system, other thalamic nuclei, and their projections into the striatum. This will allow researchers to properly define the role that the vestibular system plays in many types of striatal-mediated behaviors, associative learning and memory, and human disease models with vestibular components.

## Synthesis

Reviewing Editor: Mark Laubach, American University Decisions are customarily a result of the Reviewing Editor and the peer reviewers coming together and discussing their recommendations until a consensus is reached. When revisions are invited, a fact-based synthesis statement explaining their decision and outlining what is needed to prepare a revision will be listed below. The following reviewer(s) agreed to reveal their identity: NONE.

Thank you for sending your study to eNeuro. Two experts reviewed your manuscript and raised many concerns that will require extensive revisions to address. Please revise your manuscript to address all points below. It will be reviewed again by both reviewers before a decision will be made.

Reviewer #1

The authors used anterograde and retrograde AAV methods to identify the projections from the cortex to the striatum and from the PF to the striatum, respectively. Utilizing gerbils, which are less commonly studied in this context, and their robust auditory-evoked behavioral paradigms, the authors carefully measured and compared the striatal GFP expression from both the motor cortex and the sensory cortex. This allowed them to summarize the basic pattern of cortical projection distribution across the striatal rostro-caudal axis, elucidate PF input to the striatum, and understand how this neural circuit influences auditory discrimination tasks.

The flow of the paper is very clear. First, the authors mapped the projections from the cortex to the striatum, identifying corresponding projections from functional cortical regions. These projections were quantified using binarized fluorescent intensity. They then compared the quantified projections between dorsal and posterior regions to summarize the distribution patterns for the motor and somatosensory cortices. Additionally, they considered neural projection rules, noting that projections typically cross the corpus callosum to summarize ipsilateral and contralateral projection patterns. Following upstream anterograde tracing, the authors demonstrated that the striatum receives projections from the PF, predominantly targeting the sensory striatum, as evidenced by retrograde tracing. Besides dissecting cortical-striatal neural circuit components, the authors also conducted functional behavioral tests, suggesting that PF-striatum output might be involved in perceptive information relay by disrupting auditory processing in superior semicircular canal dehiscence models.

The authors may wish to consider the following concerns:

1. Discuss the embryonic basis for the absence of cortical projections in the ventral striatum.

2. Figure 2 C. Why are MCx and SCx colored red and ACx and VCx colored blue?

3. Cite relevant literature on the division of common subregions of the striatum in gerbils to distinguish them from other experimental rodents. Fig 2 notes that the dorsal and posterior striatum are distinguished using bregma as a landmark. What is used to distinguish other striatal regions? (i.e. dorsal vs ventral etc.) Arbitrary differences in striatal subdivisions across rodentia should be discussed as a pitfall.

4. Use 'relative proportions' to describe the comparison between ipsilateral and contralateral projections instead of 'volume'. There seems to be a reduction of intensity as well as volume.

5. Consider if n=3 is sufficient for statistical comparisons between groups.

6. Compare the distribution pattern of cortical projections to the dorsolateral and dorsomedial striatum with those observed in other experimental rodents, especially mice.

7. Add comparisons between ipsilateral and contralateral projections for PF retrograde tracing to the striatum and striatum anterograde tracing to the PF. Make sure that an important and perhaps unexpected, crossed pathway does not exist.

8. The authors state that unilateral vestibular injury impaired behavioral performance by disrupting the MVN-pf-striatal circuit, but no data shows that the injury disrupts the circuit.

9. Clarify the first paragraph of "Vestibular input to the PFN" to enhance the reader's understanding of the MVN-PF-striatum neural circuit. A diagram might be helpful.

10. Is it clear that activation of the PfN suppresses MSNs?

11. It is unclear if the authors established a "novel connection" between the medial vestibular nucleus, the Pf, and the striatum, as the circuit shown in 7A is apparently from the Allen Brain Atlas.

12. Use DAPI-indicated neuron number to confirm the quantification of fluoresce intensity.

13. Further elaborate on the preprocessing and normalization steps of the data.

14. Explain the method for choosing a baseline fluorescence intensity for each individual or clarify the approach taken if a different normalization method was used.

The authors may wish to make detailed modifications considering the following findings:

1. Decrease the contrast of all histology images for better visualization of the original anatomical structures of the brain slices (e.g., Fig 5A).

2. Add the number of subjects (n) to relevant sections.

3. In Figure 1, pair the panels (A-E, B-F, C-G, D-H) to better illustrate their corresponding relationships.

4. Figure 5B lacks distance indicators.

5. Combine Figures 7B and 7C.

6. Provide experimental data interpretation for Figure 7D.

7. Improve readability of Figure 7E. Differentiate colors in Figures 7F and 7H. The colors look the same.

8. According to Figure 7G, the correct trials percentage is above 75% by testing day 10, not 90%.

9. Figure 4A lacks significance symbols at DLS-SCx. Include statistical significance for labeling differences.

10. Add definitions for "PoM" and other abbreviations.

11. Clarify the correlation between behavioral impairment and increases in ABR threshold and c+VEMP amplitude. What is the c+VEMP amplitude?

12. Correct the typo "sneory" to "sensory."

13. Ensure consistent font size between paragraphs.

Reviewer #2

Here the authors took advantage of AAV-based viral tracing (anterograde &retrograde) to conduct and in-depth analysis of corticostriatal and thalamostriatal projections in gerbils. Compared to rodents, auditory and visual systems of gerbils are closer to humans, therefore emphasis was placed in evaluating corticostriatal projections arising from auditory and visual cortical areas. Anterograde-spreading AAVs were injected into the primary motor, somatosensory, auditory, and visual cortices, whereas retrograde AAV deliveries were performed either into striatal areas (somatosensory and auditory) or in the parafascicular nucleus.

Major comments:

(1) For a study focused on the analysis of the projection patterns for striatal afferents arising from cortical and thalamic sources, in my view here excessive emphasis was placed in studying those afferent systems coming from primary auditory and visual cortices.

(2) The title is somehow misleading by linking corticostriatal and thalamostriatal projections together with the identification of a potential route for the PF nucleus to relay vestibular information to sensorimotor striatum. Please note that in strict sense, these two parts of the title are unrelated to each other.

(3) Data from retrograde AAV deliveries into different striatal territories were only succinctly described in the Results section. Moreover, when describing corticostriatal and thalamostriatal projections, there is little sense to inject retrograde AAVs into the parafascicular nucleus.

(4) The distribution of corticostriatal and thalamostriatal projections across striatal compartments (striosomes and matrix) needs to be addressed. Such a distribution plays an important role in basal ganglia physiology.

(5) The Discussion section is a little bit thin in regards to what the results can be used for. Indeed, most of this section is biased towards discussing the relevance of vestibular inputs to the basal ganglia via the PF thalamostriatal projections.

(6) Distinctions made between the so-called "dorsal striatum" and "posterior striatum" were based on using bregma as the reference landmark. However, in the field it is by far more common to separate pre- and post-commissural striatum based on the location of the anterior commissure.

(7) Obtained data regarding patterns of corticostriatal and thalamostriatal projections in gerbils are in full keeping with what was formerly reported in rodents and in non-human primates. In this regard, a large body of literature has been neglected, as follows:

(a) Striatal afferent systems &striatal compartments:

- Graybiel 1984. Neuroscience 13:1157-1187

- Selemon &Goldman-Rakic, 1985. J Neurosci 5:776-794

- Donoghue &Herkenham 1986. Brain Res 365:397-403

- Ragsdale &Graybiel 1988. J Comp Neurol 269:506-522

- Graybiel 1990. Trends Neurosci 13:244-254

- Sadikot et al., 1992a. J Comp Neurol 315:137-159

- Sadikot et al., 1992b. J Comp Neurol 320:228-242

- Kincaid &Wilson, 1996. J Comp Neurol 374:578-592

- McFarland &Haber, 2000. J Neurosci 20:3798

- Haber et al., 2000. J Neurosci 20:2369-2382

- Borra et al., 2021. J Neurosci 41:1455-1469

(b) IT-type vs. PT-type corticostriatal-projecting neurons

- Reiner et al., 2003. J Comp Neurol 457:420-440

- Lei et al., 2004. J Neurosci 24:8289-8299

- Baillon et al., 2008. Eur J Neurosci 27:2312-2321

- Reiner et al., 2010. Front Neuroanat 4:142

- Deng et al., 2015. Front Sys Neurosci 9:51

(c) Ipsi- vs. contra-lateral corticostriatal projections

- Borra et al., 2021. J Neurosci 41:1455-1469

- Rico et al., 2024. Front Neuroanat 18:1355940

(d) thalamostriatal projections

- Dubé et al., 1988. J Comp Neurol 267:455-471

- Sidibé &Smith, 1999. Neuroscience 89:1189-1208

- Bacci et al., 2004. J Neuropathol Exp Neurol 63:20-31

- Lanciego et al., 2004. Eur J Neurosci 19:1267-1277

- Smith et al., 2004. Trends Neurosci 27:520-527

- Castle et al., 2005. J Comp Neurol 483:143-153

- Gonzalo-Martin et al., 2024. Front Neuroanat 17:1305500

Minor comments:

(1) AAV vectors:

(a) provide titration of the injected AAV suspensions

(b) AAV1.CamKII.eGFP or mCH was used for anterograde tracing purposes, whereas AAVrg.hSyn.eGFP or mCH was used for retrograde tract-tracing. The abbreviation of 'mCH' likely makes reference to mCherry, although it is a somehow unconventional abbreviation. For a better understanding, please use mCherry instead of mCH. Furthermore, the meaning of 'AAVrg' needs to be explained.

(2) Histology:

Please provide a table summarizing the list of antibodies used (primary and secondary), including commercial sources, catalogue numbers, titration used, incubation times and RRIDs

(3) Results. Whenever possible, please minimize literature references in this section.

(4) Results, section 'Patterns of corticostriatal input...' (fourth paragraph): data and values showing statistical significance should better be moved to the legend of Figures 4A &B (quite the same applies to data provided for the vestibular inputs to the PfN)

(5) Results, section 'Vestibular input to the PfN and behavioral...' In its present form, sounds like better suited for the Discussion section.

(6) Nomeclature and abbreviations for the parafascicular nucleus of the thalamus should be more consistently used throughout the manuscript (sometimes referenced as either 'Pf thalamus or PfN).

## Author Response

The review of this paper was thorough. It was clear that the reviewers were very knowledgeable of the literature in the field and the techniques used in the paper. We have revised the paper according to each critique raised. In this process we have taken the time to make thoughtful major revisions to the flow of each section. We have changed the title. The abstract and significance statements have been revised to reflect the manuscript revision where appropriate. We have bolstered the introduction with additional information and improved rationale for the study, improved the description of the methods where requested, revised the results for flow and added a limited number of retrograde analyses, moved excess information to the discussion, and refined the discussion with a main recap plus circuit diagram and two main points. The figures have been improved and rearranged to match the flow of the results section. See bolded text in the main manuscript for an overview of changes requested. The review and subsequent revision have much improved the manuscript. Below are point by point responses to each critique raised by the reviewers.

Reviewer #1 The authors used anterograde and retrograde AAV methods to identify the projections from the cortex to the striatum and from the PF to the striatum, respectively. Utilizing gerbils, which are less commonly studied in this context, and their robust auditory-evoked behavioral paradigms, the authors carefully measured and compared the striatal GFP expression from both the motor cortex and the sensory cortex. This allowed them to summarize the basic pattern of cortical projection distribution across the striatal rostro-caudal axis, elucidate PF input to the striatum, and understand how this neural circuit influences auditory discrimination tasks.

The flow of the paper is very clear. First, the authors mapped the projections from the cortex to the striatum, identifying corresponding projections from functional cortical regions. These projections were quantified using binarized fluorescent intensity. They then compared the quantified projections between dorsal and posterior regions to summarize the distribution patterns for the motor and somatosensory cortices. Additionally, they considered neural projection rules, noting that projections typically cross the corpus callosum to summarize ipsilateral and contralateral projection patterns. Following upstream anterograde tracing, the authors demonstrated that the striatum receives projections from the PF, predominantly targeting the sensory striatum, as evidenced by retrograde tracing. Besides dissecting cortical-striatal neural circuit components, the authors also conducted functional behavioral tests, suggesting that PF-striatum output might be involved in perceptive information relay by disrupting auditory processing in superior semicircular canal dehiscence models.

The authors may wish to consider the following concerns:

1. Discuss the embryonic basis for the absence of cortical projections in the ventral striatum.

We apologize for confusion based on the discussion of ventral striatum. None of the cortical regions we injected are known to project to the nucleus accumbens (e.g., Entorhinal cortex), thus our analysis focused on posterior and dorsal striatum. We believe this would be a great follow up study to continue mapping the connection between thalamus and vestibule etc.

2. Figure 2 C. Why are MCx and SCx colored red and ACx and VCx colored blue? We chose a color-blind palette since ~ 10% of men are color blind (Tol_Muted). If you printed this on an office copier the colors might have come out differently than they will appear online (or published print).

3. Cite relevant literature on the division of common subregions of the striatum in gerbils to distinguish them from other experimental rodents. Fig 2 notes that the dorsal and posterior striatum are distinguished using bregma as a landmark. What is used to distinguish other striatal regions? (i.e. dorsal vs ventral etc.) Arbitrary differences in striatal subdivisions across rodentia should be discussed as a pitfall.

The subregions in gerbil have not been identified yet (genetically/neurochemically), but the official gerbil atlas (Radtke-Schuller S 2016) delineates dorsal striatum from nucleus accumbens (core/shell).

Similarly mouse and rat have been mostly defined by their atlases into dorsal striatum and ventral striatum (NA core/shell). In addition, in mouse and rat there is strong literature on the functional organization/subdivisions of the dorsal striatum based on neurochemistry studies (patch/matrix) initially, then lesion/ephys behavioral studies (DMS/DLS Henry Yin; posterior striatum Uchida &Alloway &Tony Zador), and more recently based on tracing studies (Hinitryan Nature Neuroscience, Hunnicut elife,).

The latter studies used arbitrary cluster analysis to reveal the striatal subdivisions based on corticostriatal connectivity patterns. Based on these key studies we (and others) have largely adopted this convention for the subdivisions (Aoki elife 2019).

We have added this information into the text in the section: Patterns of corticostriatal input reveal sensorimotor compartmentalization within the gerbil striatum. 2nd paragraph.

4. Use 'relative proportions' to describe the comparison between ipsilateral and contralateral projections instead of 'volume'. There seems to be a reduction of intensity as well as volume.

We have changed any reference to volume to relative proportion.

5. Consider if n=3 is sufficient for statistical comparisons between groups.

For animal numbers we used the conventions for our previous neuroanatomical tracing papers where differences were qualitatively distinct enough that N3 (minimum) became our standard for this approach (Alloway, Smith, Watson, Mowery studies). Power analysis from these previous studies suggests N3 is a minimum for statistical reliability with this technique (g3power). Our patterns match previously published work in mice and rats, therefore we did not continue after our minimum standard was reached. In addition to this, we use Tukeys Honestly Significant Difference posthoc analysis, which accounts for variance when determining significance. This test increases confidence in statistical testing over conventional student's t-test.

6. Compare the distribution pattern of cortical projections to the dorsolateral and dorsomedial striatum with those observed in other experimental rodents, especially mice.

We have added some additional information to the discussion section: Comparative neuroanatomical innervation of the striatum in gerbil to other species.

7. Add comparisons between ipsilateral and contralateral projections for PF retrograde tracing to the striatum and striatum anterograde tracing to the PF. Make sure that an important and perhaps unexpected, crossed pathway does not exist.

Previous work from others and our own has shown that contralateral input from PF to striatum is sparse to non-existent (Castle et al., 2005; Wall NR et al., Neuron 2013; Smith JB et al., Neuron 2016). In our tissue PF contra staining in gerbil was barely above background staining if present at all. We did not include PF contra analysis because of this.

We have added a few statements to the results section: Patterns of PfN anterograde labeling reveal discrete compartmentalization and sensorimotor innervation bias within the gerbil striatum.

8. The authors state that unilateral vestibular injury impaired behavioral performance by disrupting the MVN-pf-striatal circuit, but no data shows that the injury disrupts the circuit.

We agree and have revised these statements to reflect hypothetical disruption of this circuit through asymmetrical vestibular output (c+VEMP confirmed). We propose future experiments to address this issue (e.g., chemogenetics).

9. Clarify the first paragraph of "Vestibular input to the PFN" to enhance the reader's understanding of the MVN-PF-striatum neural circuit. A diagram might be helpful.

We have clarified the circuit (1st paragraph of discussion) and added a circuit diagram (Figure 10) that provides a visual aid for the second main finding of the study.

10. Is it clear that activation of the PfN suppresses MSNs? PF is excitatory (msns) We agree this was unclear and removed the reference to this in results. We added a minor statement about this effect on the circuit in the discussion (1st paragraph of discussion, circuit diagram; Alloway 2014;2017 and Watson 2015;2018). We propose to further explore Pf/CM/PoM interactions with each other and circuit in regard to vestibular input.

11. It is unclear if the authors established a "novel connection" between the medial vestibular nucleus, the Pf, and the striatum, as the circuit shown in 7A is apparently from the Allen Brain Atlas.

We agree. This circuit has been established by Henry Yin's group in mouse (Watson 2021) and is publicly available in Allen Brain Atlas. We have removed reference to it being novel, and changed these statements to reflect a verification of this pathway in gerbil.

12. Use DAPI-indicated neuron number to confirm the quantification of fluoresce intensity.

We have updated Figure 1 (left panel). The fluorescence staining is a mixture of drivers and modulators onto cell bodies and fibers of passage (with and without synaptic bifurcation). For this gross anatomical study, we used a non-descriptive method to quantify labeling, but we do plan to revisit the main findings with more descript methods that allow comparative analysis between the types of synaptic innervation indirectly revealed by the fluorescent imaging. This will include higher magnification cell/synapse counting and quantitative/qualitative analysis.

13. Further elaborate on the preprocessing and normalization steps of the data & 14. Explain the method for choosing a baseline fluorescence intensity for each individual or clarify the approach taken if a different normalization method was used.

We have made significant revisions to the methods section. This includes explicit preprocessing and normalization methods. Methods: AAV Fluorescence Quantification The authors may wish to make detailed modifications considering the following findings:

1. Decrease the contrast of all histology images for better visualization of the original anatomical structures of the brain slices (e.g., Fig 5A).

We have added coronal anatomical outlines for reference in each figure with indicators for injection placement as well as stereotaxic coordinates (gerbil atlas).

2. Add the number of subjects (n) to relevant sections.

N for each experiment is now included in each results section.

3. In Figure 1, pair the panels (A-E, B-F, C-G, D-H) to better illustrate their corresponding relationships.

We have revised Figure 1 (now Figure 3) in this convention (Cortical Injection paired with Thalamic Labeling).

4. Figure 5B lacks distance indicators.

We have added distance indicators. Now Figure 7A. Distance indicators have been added anywhere appropriate throughout the figures.

5. Combine Figures 7B and 7C.

This is now figure 8. We combined B&C.

6. Provide experimental data interpretation for Figure 7D.

This is now Figure 8. We have added details for data interpretation D to the results section.

7. Improve readability of Figure 7E. Differentiate colors in Figures 7F and 7H. The colors look the same.

This is now figure 9. We have made the data figure larger and improved the readability of this figure.

8. According to Figure 7G, the correct trials percentage is above 75% by testing day 10, not 90%.

This is now Figure 6. We have changed this to 75%.

9. Figure 4A lacks significance symbols at DLS-SCx. Include statistical significance for labeling differences.

These have been added.

10. Add definitions for "PoM" and other abbreviations.

All acronyms should now have corresponding definitions at least once per section.

11. Clarify the correlation between behavioral impairment and increases in ABR threshold and c+VEMP amplitude. What is the c+VEMP amplitude? We have added clarifying statements to this section.

12. Correct the typo "sneory" to "sensory." Corrected the typo 13. Ensure consistent font size between paragraphs.

Font size has been set to Arial 11 throughout the manuscript.

Reviewer #2 Here the authors took advantage of AAV-based viral tracing (anterograde &retrograde) to conduct and in-depth analysis of corticostriatal and thalamostriatal projections in gerbils. Compared to rodents, auditory and visual systems of gerbils are closer to humans, therefore emphasis was placed in evaluating corticostriatal projections arising from auditory and visual cortical areas. Anterograde-spreading AAVs were injected into the primary motor, somatosensory, auditory, and visual cortices, whereas retrograde AAV deliveries were performed either into striatal areas (somatosensory and auditory) or in the parafascicular nucleus.

Major comments: (1) For a study focused on the analysis of the projection patterns for striatal afferents arising from cortical and thalamic sources, in my view here excessive emphasis was placed in studying those afferent systems coming from primary auditory and visual cortices.

My lab focuses on Auditory and Vestibular research. The study started out as an exploration of posterior striatum and vestibular to PfN to posterior striatum inputs. Somatosensory and motor experiments were added later in the study for completeness. While revising the paper we have kept this in mind and tried to balance emphasis across cortical systems with a core focus on PfN innervation along the striatal AP axis and Dorsal/Posterior labeling. (2) The title is somehow misleading by linking corticostriatal and thalamostriatal projections together with the identification of a potential route for the PF nucleus to relay vestibular information to sensorimotor striatum. Please note that in strict sense, these two parts of the title are unrelated to each other.

We have changed the title to more accurately reflect the main findings of the study: Neuroanatomical mapping of gerbil corticostriatal and thalamostriatal projections reveals the parafascicular nucleus as a relay for vestibular information to the entire striatum (3) Data from retrograde AAV deliveries into different striatal territories were only succinctly described in the Results section. Moreover, when describing corticostriatal and thalamostriatal projections, there is little sense to inject retrograde AAVs into the parafascicular nucleus.

We have reordered the results section for clarity and flow. Retrograde striatal tracing was initially done to parse out the relative compartmentalization of the posterior striatum. This revealed a somatosensory biased region more anterior (in the PDS) and an auditory/visual biased region more posterior (in the PDS). We added some more thalamic analyses here as well. We also noted the bias in PfN labeling between these two injection sites in the A/P plane. These motivated all of the rest of the anterograde tracing. The only reason we injected retrograde tracer into PF was to verify the long range projection from medial vestibular nucleus that motivated results and Figures for 8,9,10. (4) The distribution of corticostriatal and thalamostriatal projections across striatal compartments (striosomes and matrix) needs to be addressed. Such a distribution plays an important role in basal ganglia physiology.

We have added detailed information to the discussion: Comparative neuroanatomical innervation of the striatum in gerbil to other species 1st and 3rd paragraph. Suggested a limitation and future experiment. (5) The Discussion section is a little bit thin in regards to what the results can be used for. Indeed, most of this section is biased towards discussing the relevance of vestibular inputs to the basal ganglia via the PF thalamostriatal projections.

We have revised the paper to emphasis this in three ways. 1st the gerbil is an excellent animal model to use for vestibular based research and will be useful for exploring the vestibular circuits that connect with striatum. We have added some information throughout the discussion and a small amount in the introduction. 2nd we have added details for future experiments that can be carried out by our lab and others in the field (in mice and rats etc) in the first paragraph of the discussion and the vestibular striatum section of the discussion. 3rd we have included a circuit diagram to facilitate interpretation of the results and spur experimental designs that would explore the circuit. The addition of vestibular input to the vast body of striatal research will open up a new area of focus for animal research in regards to basic science (functional circuits/models) and disease/disorders (e.g., Parkinson's). (6) Distinctions made between the so-called "dorsal striatum" and "posterior striatum" were based on using bregma as the reference landmark. However, in the field it is by far more common to separate pre- and post-commissural striatum based on the location of the anterior commissure.

We agree. We used Bregma during the surgery for the stereological injection sites, but the anterior commissure is what was used to estimate bregma histologically for separation and analysis (dorsal vs posterior striatum). We have revised the language in the paper and figures. (7) Obtained data regarding patterns of corticostriatal and thalamostriatal projections in gerbils are in full keeping with what was formerly reported in rodents and in non-human primates. In this regard, a large body of literature has been neglected, as follows: (a) Striatal afferent systems &striatal compartments:

- Graybiel 1984. Neuroscience 13:1157-1187 - Selemon &Goldman-Rakic, 1985. J Neurosci 5:776-794 - Donoghue &Herkenham 1986. Brain Res 365:397-403 - Ragsdale &Graybiel 1988. J Comp Neurol 269:506-522 - Graybiel 1990. Trends Neurosci 13:244-254 - Sadikot et al., 1992a. J Comp Neurol 315:137-159 - Sadikot et al., 1992b. J Comp Neurol 320:228-242 - Kincaid &Wilson, 1996. J Comp Neurol 374:578-592 - McFarland &Haber, 2000. J Neurosci 20:3798 - Haber et al., 2000. J Neurosci 20:2369-2382 - Borra et al., 2021. J Neurosci 41:1455-1469 (b) IT-type vs. PT-type corticostriatal-projecting neurons - Reiner et al., 2003. J Comp Neurol 457:420-440 - Lei et al., 2004. J Neurosci 24:8289-8299 - Baillon et al., 2008. Eur J Neurosci 27:2312-2321 - Reiner et al., 2010. Front Neuroanat 4:142 - Deng et al., 2015. Front Sys Neurosci 9:51 (c) Ipsi- vs. contra-lateral corticostriatal projections - Borra et al., 2021. J Neurosci 41:1455-1469 - Rico et al., 2024. Front Neuroanat 18:1355940 (d) thalamostriatal projections - Dubé et al., 1988. J Comp Neurol 267:455-471 - Sidibé &Smith, 1999. Neuroscience 89:1189-1208 - Bacci et al., 2004. J Neuropathol Exp Neurol 63:20-31 - Lanciego et al., 2004. Eur J Neurosci 19:1267-1277 - Smith et al., 2004. Trends Neurosci 27:520-527 - Castle et al., 2005. J Comp Neurol 483:143-153 - Gonzalo-Martin et al., 2024. Front Neuroanat 17:1305500 We appreciate the addition of this body of literature and have included it throughout the introduction and especially discussion where appropriate (mostly Comparative neuroanatomical innervation of the striatum in gerbil to other species) We have also added additional references and information to further bolster the review and anatomical comparisons between species.

Minor comments: (1) AAV vectors: (a) provide titration of the injected AAV suspensions We added this to the methods. These are commercially sourced from ADDgene and have titers on each vial. (b) AAV1.CamKII.eGFP or mCH was used for anterograde tracing purposes, whereas AAVrg.hSyn.eGFP or mCH was used for retrograde tract-tracing. The abbreviation of 'mCH' likely makes reference to mCherry, although it is a somehow unconventional abbreviation. For a better understanding, please use mCherry instead of mCH. Furthermore, the meaning of 'AAVrg' needs to be explained.

We added the full definition for mCherry and defined rg (retrograde) and rationale for CamKII vs hSyn promoters. (2) Histology:

Please provide a table summarizing the list of antibodies used (primary and secondary), including commercial sources, catalogue numbers, titration used, incubation times and RRIDs We didn't use any antibodies. These were all viral fluorescent protein expressions (green fluorescent protein or mCherry fluorescent (red) protein. We have included all AAV vectors and transgene expression times (all 3 weeks) used in the methods section: AAV Vectors (3) Results. Whenever possible, please minimize literature references in this section.

We moved referenced material to the discussion sections where appropriate to minimize discussion material in the results section. (4) Results, section 'Patterns of corticostriatal input...' (fourth paragraph): data and values showing statistical significance should better be moved to the legend of Figures 4A &B (quite the same applies to data provided for the vestibular inputs to the PfN) We have reduced the bulky stats (data dumps) in the main text by moving them to table 1&2. (5) Results, section 'Vestibular input to the PfN and behavioral...' In its present form, sounds like better suited for the Discussion section.

We moved discussion material to Discussion: Is there a compartmentalized vestibular representation in striatum? (6) Nomeclature and abbreviations for the parafascicular nucleus of the thalamus should be more consistently used throughout the manuscript (sometimes referenced as either 'Pf thalamus or PfN).] PfN is now used throughout the manuscript and figures.
