# Supplementary figures and images for: Neuroanatomical Mapping of Gerbil Corticostriatal and Thalamostriatal Projections Reveals the Parafascicular Nucleus as a Relay for Vestibular Information to the Entire Striatum
Source: eNeuro. 2025 Mar 7;12(3):ENEURO.0246-24.2025. doi: 10.1523/ENEURO.0246-24.2025 (PMC11913323; doi:10.1523/ENEURO.0246-24.2025)

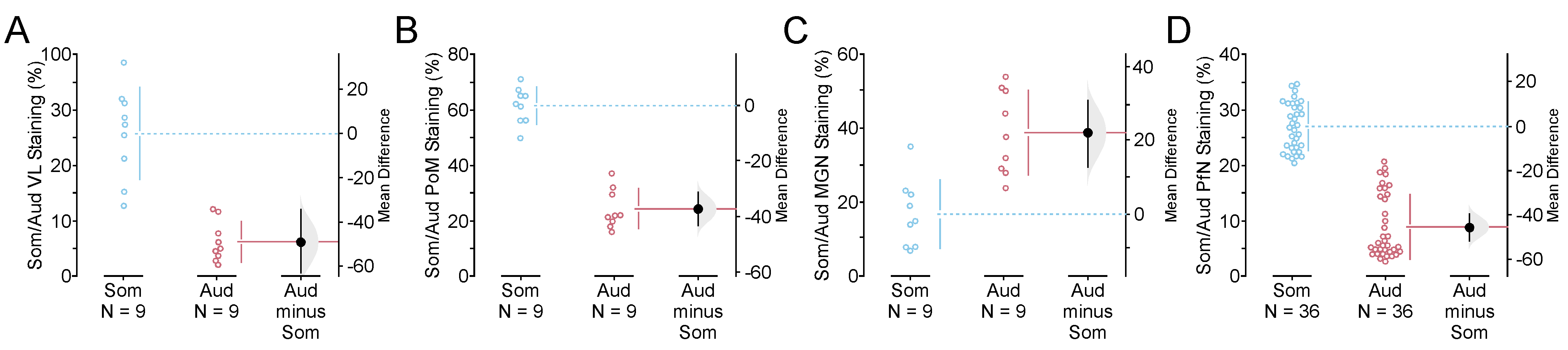

Supplement: Figure 2-1 — Estimated Mean Differences for retrograde labeling of thalamic nuclei after injection into somatosensory or auditory striatum. A) A Gardner-Altman estimation plot showing the mean difference between VL labeling for somatosensory striatum injections versus auditory striatum injections. Both groups are plotted on the left axes; the mean difference is plotted on a floating axes on the right as a bootstrap sampling distribution. The mean difference is depicted as a dot; the 95% confidence interval is indicated by the ends of the vertical error bar; students t-test (t = 6.5), p < 0.001. B) A Gardner-Altman estimation plot showing the mean difference between PoM labeling for somatosensory striatum injections versus auditory striatum injections. Conventions are the same as in A; students t-test (t = 11.7), p < 0.001. C) A Gardner-Altman estimation plot showing the mean difference between MGN labeling for somatosensory striatum injections versus auditory striatum injections. Conventions are the same as in A; students t-test (t = -4.5), p < 0.01. D) A Gardner-Altman estimation plot showing the mean difference between PfN labeling for somatosensory striatum injections versus auditory striatum injections. Conventions are the same as in A; students t-test (t = 15.1), p < 0.001. Download Figure 2-1, TIF file. [file eneuro-12-ENEURO.0246-24.2025-s002.tif]

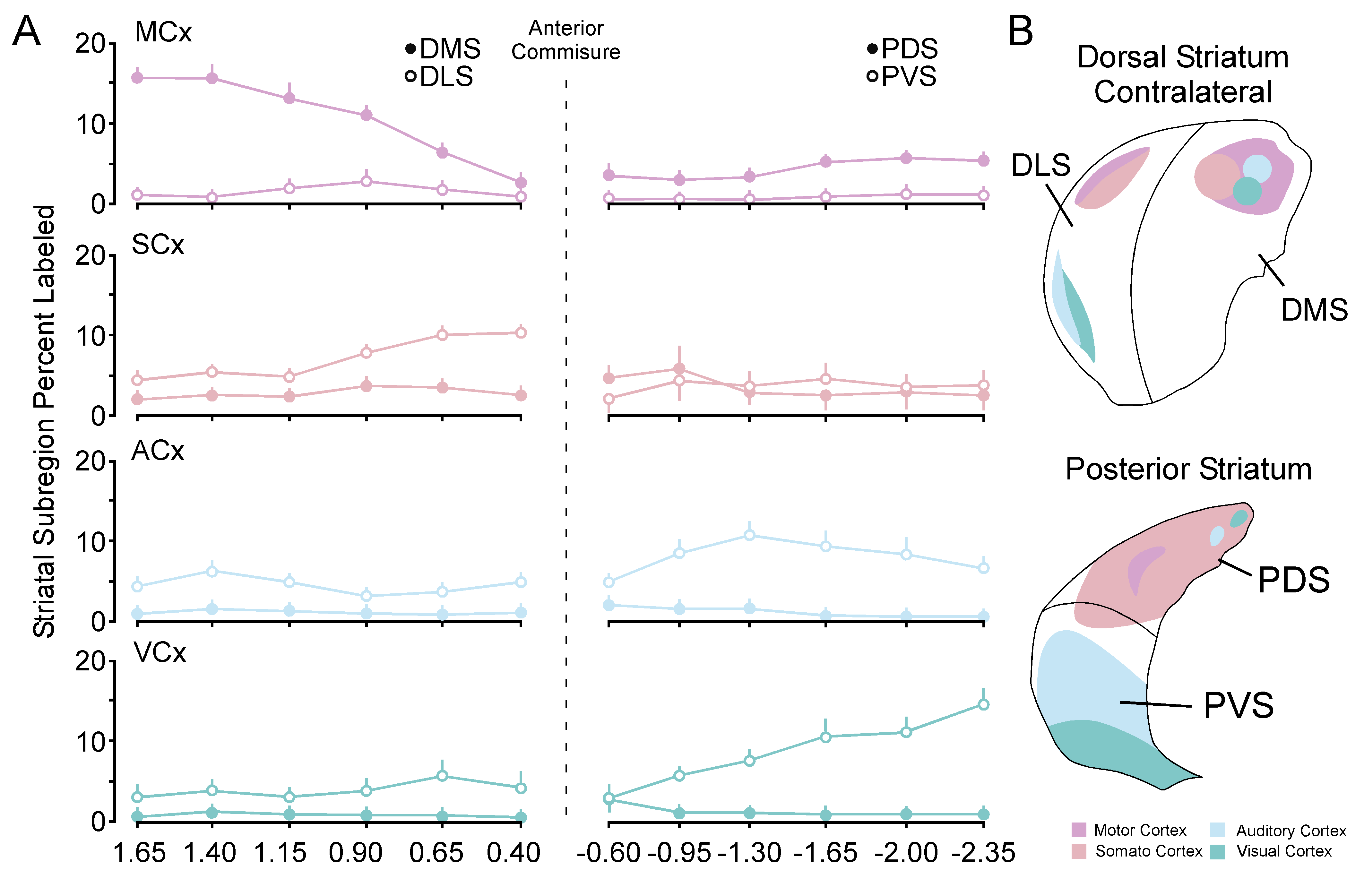

Supplement: Figure 6-1 — Quantification of contralateral mediolateral labeling of the dorsal striatum and dorsoventral labeling of the posterior striatum. A) Line plots showing mediolateral or dorsoventral comparisons of the average labeling at each stereotaxic coordinate over the dorsal (left) and posterior (right) A/P axis of the striatum contralateral to injection sites. Data was separated into dorsomedial and dorsolateral sub compartments in the dorsal striatum and dorsal and ventral sub compartments for the posterior striatum for MCx, SCx, ACx, and VCx corticostriatal labeling. In the contralateral dorsal striatum, comparing medial to lateral sub compartment labeling revealed significantly more labeling in the DMS for motor inputs (MCx DMS vs MCx DLS; F[1,4] = 21.3, p < 0.001), more balanced labeling between somatosensory inputs with slightly more labeling in DLS (SCx DMS vs SCx DLS; F[1,4] = 11.3, p < 0.01), and significantly more labeling in DLS for auditory and visual inputs (ACx DMS vs ACx DLS; F[1,4] = 28.6, p < 0.001; VCx DMS vs VCx DLS; F[1,4] = 9.3, p < 0.05). For posterior striatum, there was significantly more labeling for motor inputs in the PDS (MCx PDS vs MCx PVS; F[1,4] = 8.9, p < 0.01), non-significant balanced labeling for somatosensory inputs between dorsal and ventral sub compartments (SCx PDS vs SCx PVS; F[1,4] = 1.6, p = 0.95), and significantly more labeling in the PVS for auditory and visual inputs (ACx PDS vs ACx PVS; F[1,4] = 35.9, p < 0.001; VCx PDS vs VCx PVS; F[1,4] = 12.5, p < 0.01). Labeling reveals sub compartment bias as well as compartmentalization (peaks) along the A/P axis for each striatal region that correspond to those reported for ipsilateral labeling. B) Diagram showing the general pattern of contralateral labeling for motor, somatosensory, auditory, and visual cortex in the dorsal and posterior striatum. Dorsomedial Striatum, DMS; Dorsolateral striatum, DLS; Posterior Dorsal Striatum, PDS; Posterior Ventral Striatum, PVS; Motor Cortex, MCx; Somat [file eneuro-12-ENEURO.0246-24.2025-s003.tif]

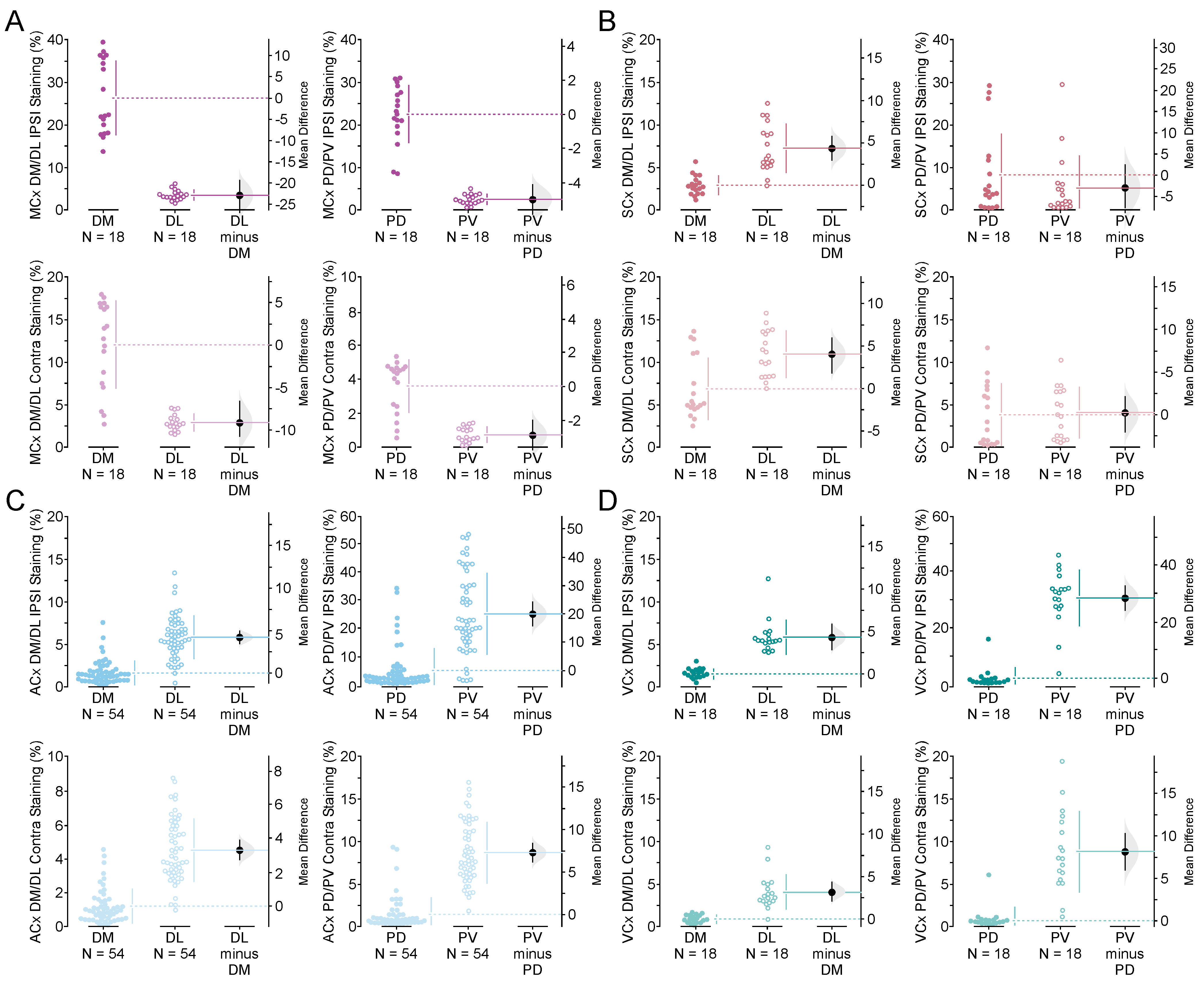

Supplement: Figure 6-2 — Estimated Mean Differences for MCx, SCx, ACx, and VCx corticostriatal striatum sub compartmental labeling in dorsal and posterior striatum. A) A Gardner-Altman estimation plot showing the mean difference between MCx labeling for labeling between DMS and DLS in dorsal striatum (left) and PDS and PVS in posterior striatum (right) for ipsilateral (top) and contralateral (bottom) labeled sections. For each estimation plot both groups are plotted on the left axes; the mean difference is plotted on a floating axes on the right as a bootstrap sampling distribution. The mean difference is depicted as a dot; the 95% confidence interval is indicated by the ends of the vertical error bar; MCx DMS vs DLS Ipsilateral students t-test (t = 11.08), p < 0.001; MCx PDS vs PVS Ipsilateral students t-test (t = 12.55), p < 0.001; MCx DMS vs DLS Contralateral students t-test (t = 7.39), p < 0.001; MCx PDS vs PVS Contralateral students t-test (t = 7.63), p < 0.001. B) A Gardner-Altman estimation plot showing the mean difference between SCx labeling for labeling between DMS and DLS in dorsal striatum (left) and PDS and PVS in posterior striatum (right) for ipsilateral (top) and contralateral (bottom) labeled sections. Conventions are the same as in A; SCx DMS vs DLS Ipsilateral students t-test (t = -3.79), p < 0.01; SCx PDS vs PVS Ipsilateral students t-test (t = 1.05), p = 0.30; SCx DMS vs DLS Contralateral students t-test (t = -3.08), p < 0.01; SCx PDS vs PVS Contralateral students t-test (t = -0.15), p = 0.88.C) A Gardner-Altman estimation plot showing the mean difference between ACx labeling for labeling between DMS and DLS in dorsal striatum (left) and PDS and PVS in posterior striatum (right) for ipsilateral (top) and contralateral (bottom) labeled sections. Conventions are the same as in A; ACx DMS vs DLS Ipsilateral students t-test (t = -10.01), p < 0.001; ACx PDS vs PVS Ipsilateral students t-test (t = -9.16), p < 0.001; ACx DMS vs DLS Contralateral students t-test (t = -11.77), p [file eneuro-12-ENEURO.0246-24.2025-s004.tif]

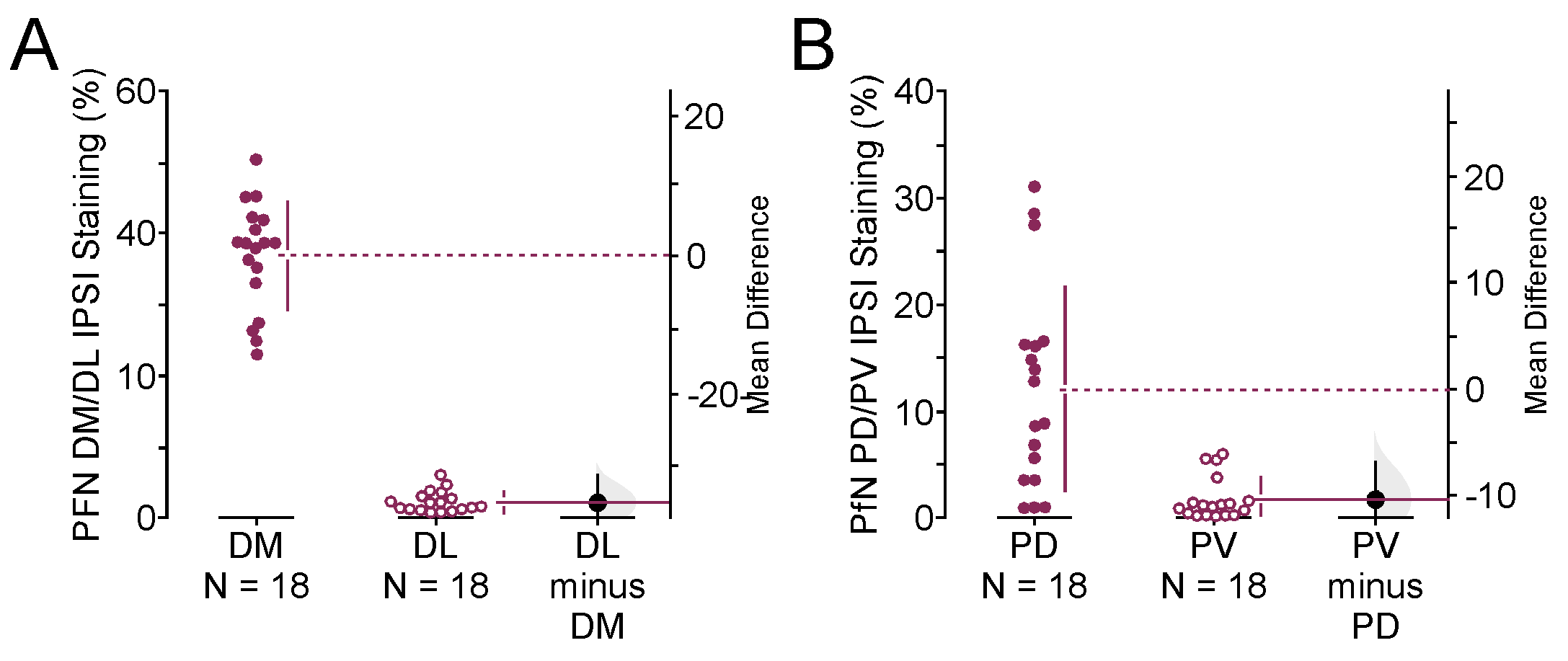

Supplement: Figure 7-1 — Estimated Mean Differences for PfN thalamostriatal striatum sub compartmental labeling in dorsal and posterior striatum. A) A Gardner-Altman estimation plot showing the mean difference between PfN labeling for labeling between DMS and DLS in dorsal striatum. For each estimation plot both groups are plotted on the left axes; the mean difference is plotted on a floating axes on the right as a bootstrap sampling distribution. The mean difference is depicted as a dot; the 95% confidence interval is indicated by the ends of the vertical error bar; PfN DMS vs DLS students t-test (t = 19.32), p < 0.001. B) A Gardner-Altman estimation plot showing the mean difference between PfN labeling between PDS and PVS in posterior striatum. Conventions are the same as in A; students t-test (t = 4.51), p < 0.001. Dorsomedial Striatum, DMS; Dorsolateral striatum, DLS; Posterior Dorsal Striatum, PDS; Posterior Ventral Striatum, PfN; Parafascicular Nucleus. Download Figure 7-1, TIF file. [file eneuro-12-ENEURO.0246-24.2025-s005.tif]
